# The impact of explaining vegetarian meal requests on the affective responses and perceptions of meat eaters

**DOI:** 10.1038/s41598-024-74479-1

**Published:** 2024-10-16

**Authors:** Kate Laffan, Emma Howard

**Affiliations:** 1 The London School of Economics and Political Science, London, UK; 2 Technological University, Dublin, Ireland

**Keywords:** Meat restrictor, Vegetarian, Social situations, Perceptions, Affective responses, Human behaviour, Psychology

## Abstract

Social situations can present challenges for those looking to reduce or avoid eating meat. In an experimental vignette study with a large representative sample of 1,117 UK-based meat eaters, we explore affective responses to requests for vegetarian meals and perceptions of those making the request across four social situations: a BBQ, a pub, a restaurant dinner, and a dinner party. We examine moderators of these perceptions including whether the requester presents themselves as vegetarian or a meat restrictor and whether they cite health, environmental or no motives for their request. Overall, we find that these moderators have a greater impact on meat eaters’ perceptions of the requester than on their affective responses to the request. We also find that these outcomes are more impacted by the mention of motives than they are diet type. Standout findings include evidence that people citing environmental motives for their vegetarian meal request are perceived more negatively, while those with health motives are perceived more positively. We also identify substantial heterogeneity across the respondents’ levels of meat attachment: those who are more attached to meat have more negative perceptions of vegetarian meal requesters and experience higher negative affect and lower positive affect in response to the request.

**Supplementary Information:**

The online version contains supplementary material available at 10.1038/s41598-024-74479-1.

## Introduction

The average Western diet is currently characterised by overconsumption of meat^1^. Sustainable dietary shifts towards people reducing their meat consumption can help promote both planetary and individual health^2^. Global food production accounts for 35% of all carbon emissions: most of these emissions are from animal-based food production which accounts for twice the emissions of plant-based food overall^3,4^. Consequently, reducing meat consumption is one of the most efficient and cost-effective ways individuals can reduce their carbon footprint^5,6^. Reduced meat consumption also has significant health benefits, including lower obesity and mortality risk^7–10^.

Despite the significant benefits of reductions in meat consumption, meat is embedded in Western culture and features strongly in everyday food consumption^11,12^. A recent survey of 24 European countries found on average that over 90% of respondents ate meat, ranging from 95% in Hungary and the Czech Republic to 85% in the UK and Ireland^13^. It is also widely socially acceptable to eat meat^14^. Research highlights the important influence of these social norms on people’s meat consumption, both in terms of what others in relevant reference groups eat (descriptive norms) and think people ought to eat (injunctive norms)^15^. Although there are positive trends in both behaviour and attitudes towards, and support for reducing meat consumption^16–18^, those abstaining or intentionally restricting their meat consumption are still a minority^19^.

The cultural embeddedness of meat consumption, as well as the existence of meat-centric social norms, can result in conflict for those who do not want to eat meat. Vegetarians and vegans report both expecting to experience and experiencing negativity from others stemming from their dietary choices^20,21^. Such negative responses can act as an impediment to people making non-meat choices: there is evidence suggesting that some vegans and vegetarians will eat meat to avoid social conflict and make social situations flow more smoothly^22,23^ and pro-meat norms have been shown to inhibit members of these groups from expressing meat-free preferences^24^. Returning to meat consumption after attempting to follow a meat-free diet is also correlated with a reported lack of social support for diet choice^25^.

Not all meat abstainers are viewed equally, however. There is emerging evidence that the perceptions of those abstaining from meat vary depending on their reported diet – e.g., meat restrictor, vegetarian or vegan. For example, researchers in Brazil found that people were more supportive of those reducing animal-based products rather than eliminating them from diets^26^. Additionally, a study in the Netherlands demonstrated that a dynamic communication style around dietary shifts away from meat, stressing a gradual transition to the diet and struggles adhering to it, induced less moral threat and fewer negative perceptions of the individual than a static communication style, emphasising the ease and permanence of the diet^16^. Relatedly, in the UK, university students and staff rated meat restrictors more favourably than vegetarians and habitual meat eaters, although respondents generally held favourable attitudes towards vegetarians^27^.

Research has shown that the motives behind people’s diets also influence how meat abstainers are perceived. In the Brazilian study cited above, those with non-moral motives for refusing meat were perceived as less arrogant and threatening than those with moral motivations^26^. Similar results were documented in a Dutch sample which considered the diets of vegetarians and vegans^16^. These patterns are thought to be explained in part by the relative difference in the level of moral threat, i.e., the challenge to the meat eaters’ sense of moral identity, that abstaining from meat for these different reasons poses^28^. They may also evoke ethical dissonance in those who share moral values and recognise the need to change their diet but have not done so^29^.

While these studies offer initial evidence of how responses to and perceptions of meat abstainers vary according to their cited diets and motives, they tend not to examine the direct responses to requests for non-meat options in meal scenarios and do not consider how providing some explanation (be it diet or motivation) compares to providing none at all.

In the current work, we use an experimental vignette approach to investigate the affective responses and perceptions of a representative sample of the UK population of meat eaters towards individuals who request or order a vegetarian meal across four common meal scenarios in the UK – a pub carvery, a restaurant dinner, a dinner party and a BBQ at home. This approach allows us to explore the responses (both in terms of perceptions and affective responses) facing those seeking to abstain from meat consumption in social situations who offer either no explanation, or mention one of two dietary lifestyles (meat restrictor or vegetarian), or mention a dietary choice and one of two motivations (environmental or health). In colloquial terms, we examine whether when making vegetarian meal requests if you are explaining, are you losing?

We expect meat eaters to respond more favourably to people who make a vegetarian meal request as part of their efforts to limit their meat consumption, as opposed to those following a vegetarian diet. This hypothesis is based on the existing evidence that indicates that people often respond negatively to and have poor perceptions of people who follow strict meat-free diets. Our first hypothesis is as follows:

H1: Meat eaters’ responses will be more positive, in terms of both perceptions and affect, when a request for a vegetarian meal is explained based on the requester limiting their meat consumption, compared to when no explanation is given. Conversely, meat eaters’ responses will be less positive, in terms of both perceptions and affect, when a request for a vegetarian meal is explained based on the requester following a vegetarian diet, compared to when no explanation is given.

We additionally expect the perceptions of and emotional responses to meat abstainers to differ depending on whether and how the dietary choice is explained in terms of motives. Health motives are most directly associated with personal benefits and concern for one’s own wellbeing, whereas environmental motives are linked to pro-social behaviour where the benefits extend beyond the individual^30^. Meat eaters may react more positively when individuals explain they are abstaining from meat for health reasons, compared to when no reason is given, as personal reasons do not infer any responsibility on the meat eaters to also abstain from meat. In contrast, mentioning environmental reasons may evoke moral threat and cause ethical dissonance in some^29^. Our second hypothesis, therefore, is:

H2: Meat eaters’ responses will be more positive, in terms of both perceptions of the meat abstainer and affect in the moment, when a request for a vegetarian meal is explained based on health motives, compared to when no explanation is given. Conversely, meat eaters’ responses will be less positive, in terms of both perceptions of the meat abstainer and affect in the moment, when a request for a vegetarian meal is explained based on environmental motives, compared to when no explanation is given.

Meat eaters have been shown to vary in terms of their bond to meat consumption, or ‘meat attachment’^31^. We expect those with higher levels of meat attachment to have more negative responses to others requesting vegetarian meals, compared to their less attached counterparts. Our third hypothesis is:

H3: Meat eaters with high levels of meat attachment will respond less positively to vegetarian requests, in terms of perceptions of the meat abstainer and affect in the moment, compared with those with relatively lower meat attachment.

Finally, meat eaters might view people who report following a vegetarian diet as more of an outgroup in terms of meat consumption (and implicitly attachment) than meat restrictors and respond less positively to them. At the same time, the behaviour of vegetarians may feel distant to those with high meat attachment, whereas that of restrictors less so. Insofar as the latter threatens the attached meat eaters’ stance on meat more, they may respond more negatively to a meat restrictor than a vegetarian. We would also expect those with high meat attachment to respond particularly badly to any reason being cited for a vegetarian request, given the implicit negative information regarding behaviour they are attached to, however, it is unclear whether health or environmental motives would be more negatively received. We therefore also include exploratory analysis of the potential interactions between meat attachment, diets and motives.

In addition to examining our formal hypotheses, we include further exploratory analysis which investigates whether perceptions and affective responses vary across the location and company associated with the meal scenarios. We also explore expected levels of comfort for both the meat eater and vegetarian meal requester, whether meat eaters responsible for catering say they would accommodate requests for vegetarian meals, and the likelihood of the meal requester being invited to a similar occasion in future.

In providing evidence on the above questions, our analysis helps to shed light on the social responses facing people abstaining from meat across different meal scenarios, highlighting potential barriers to them making the request in the first place and pursuing sustainable dietary shifts more generally.

## Results

### The impact of mentioning dietary restrictions

We conduct a multilevel analysis of respondents’ perceptions of and affective responses to vegetarian meal requests across four social meal settings, identifying within-person variation in these variables across diet types (vegetarian or meat restrictor). Our first pre-registered hypothesis (H1) is that meat eaters will respond more favourably to people who make a vegetarian meal request as part of their efforts to limit their meat consumption, and less favourably to those who request because they follow a vegetarian diet. The hypothesis is partly supported by the results of our analysis; we find no significant differences in perceptions of the requester across diet types, but we do find that meat restrictors elicit some positive affective responses.

**Fig. 1:**
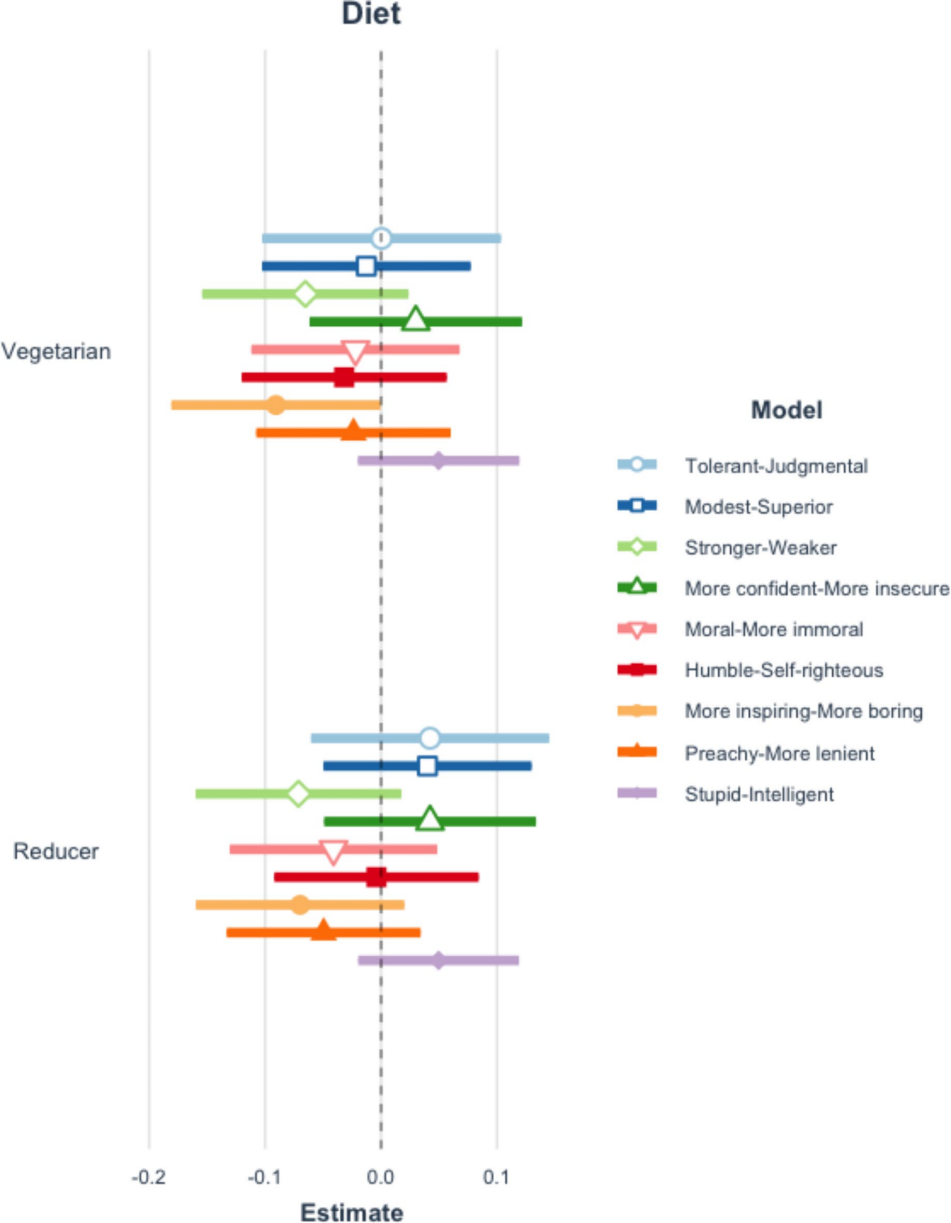
Coefficient plot of perceptions and diet mentioned. This figure reflects 9 separate multilevel models which examine the impact of mentioning a diet type on the perceptions of the requester. Error bars represent the 99% confidence intervals of estimates. Table A10 in the appendix presents the full results of all estimated models. The vegetarian-inspiring estimate is not significant when Benjamini-Hochberg adjustments are applied.

**Fig. 2:**
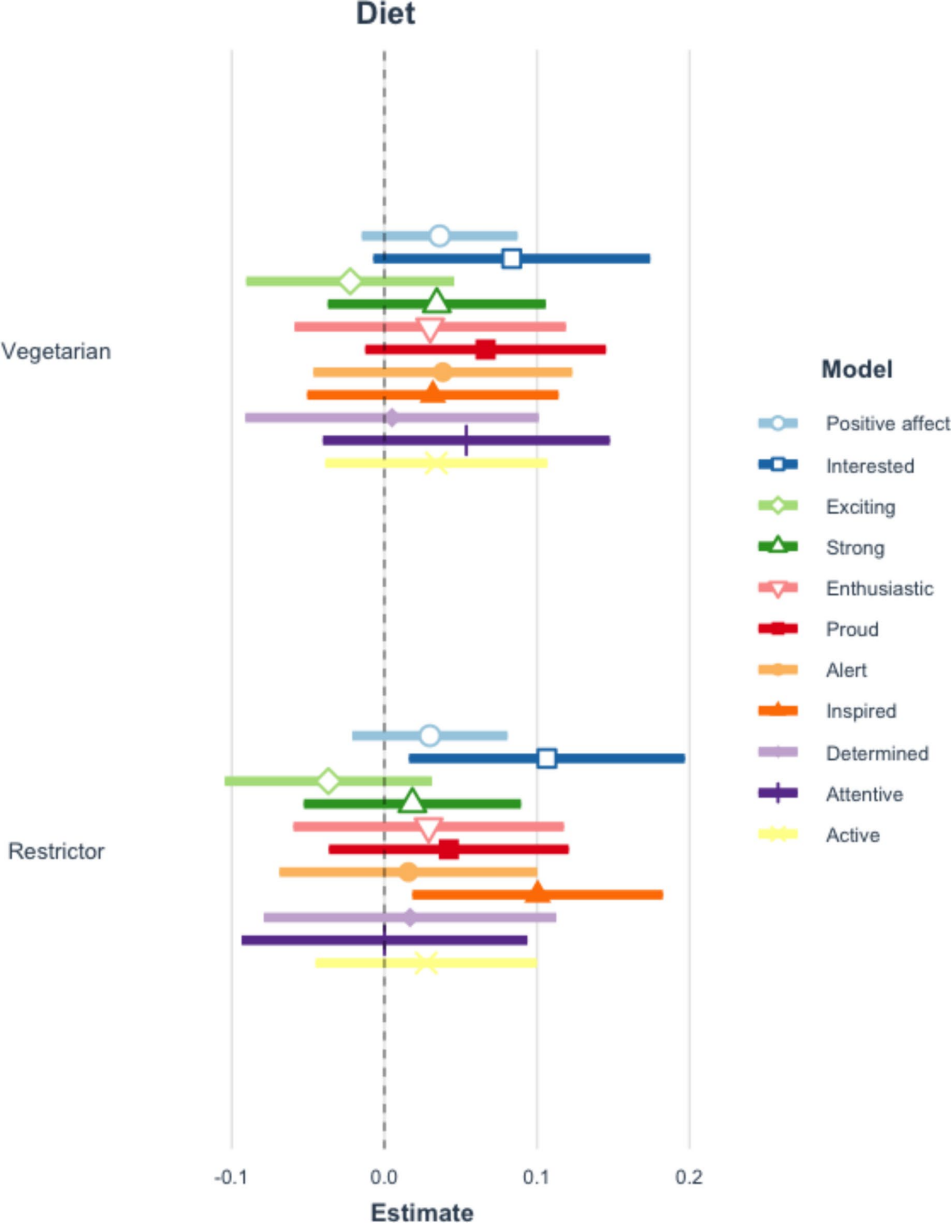
Coefficient plot of positive affect and diet mentioned. This figure reflects 11 separate multilevel models which examine the impact of mentioning a diet type on positive affective responses. Error bars represent 99% confidence intervals of estimates. Table A11 in the appendix presents the full results of all estimated models.

**Fig. 3:**
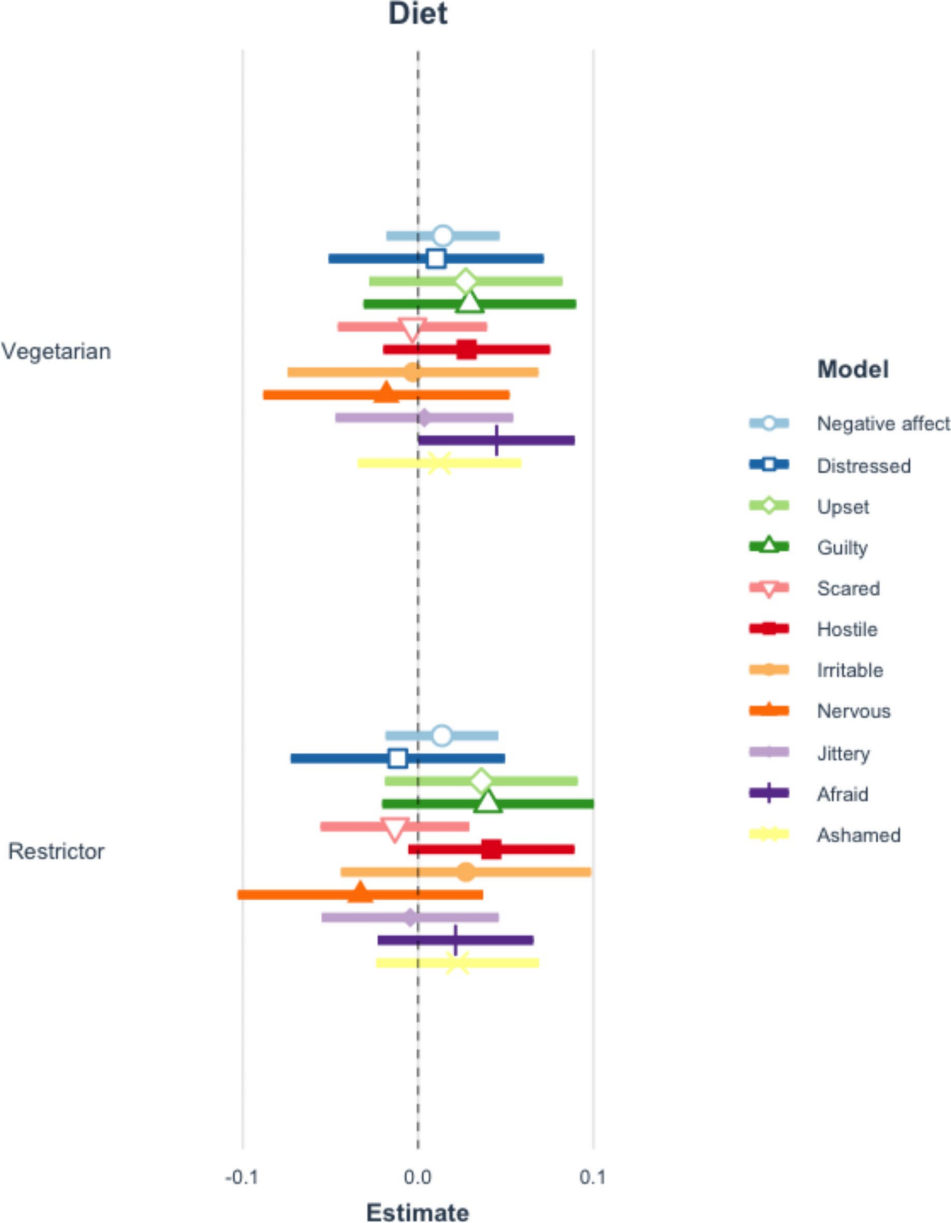
Coefficient plot of negative affect and diet mentioned. This figure reflects 11 separate multilevel models which examine the impact of mentioning a diet type on negative affective responses. Error bars represent 99% confidence intervals of estimates. Table A12 in the appendix presents the full results of all estimated models. The vegetarian-afraid estimate is not significant when Benjamini-Hochberg adjustments are applied.

Applying the Benjamini-Hochberg procedure to correct for repeated testing, there are no significant differences in the perceptions of requesters by diet type. See Fig. 1 and Table A10 of the Appendix. Additionally, there are no statistically significant relationships between either positive or negative affect overall and diet type (restrictor or vegetarian). However, meat eaters are more interested and inspired when an individual requests a vegetarian meal option and mentions that they are restricting their meat consumption, compared to when no diet is stated. See Figs. 2 and 3 and Tables A11-12 of the Appendix.

While the evidence suggests that, contrary to our hypothesis, there are no different perceptions of the two diet types, there are positive emotional responses to restrictors. People feel more interested and inspired when the requestor explains that they are restricting their meat consumption.

### The impact of mentioning motives

We conduct a multilevel analysis of respondents’ perceptions of and affective responses to vegetarian meal requests across four social meal settings, identifying within-person variation in these variables across motivations (health or environmental). Our second pre-registered hypothesis (H2) is that meat eaters will respond more favourably to people who cite health reasons for their diet, and less favourably to those who cite environmental reasons when requesting a vegetarian meal. The results for both perceptions and affective responses are more favourable towards vegetarian meal requestors with health motives and largely support this hypothesis.

**Fig. 4:**
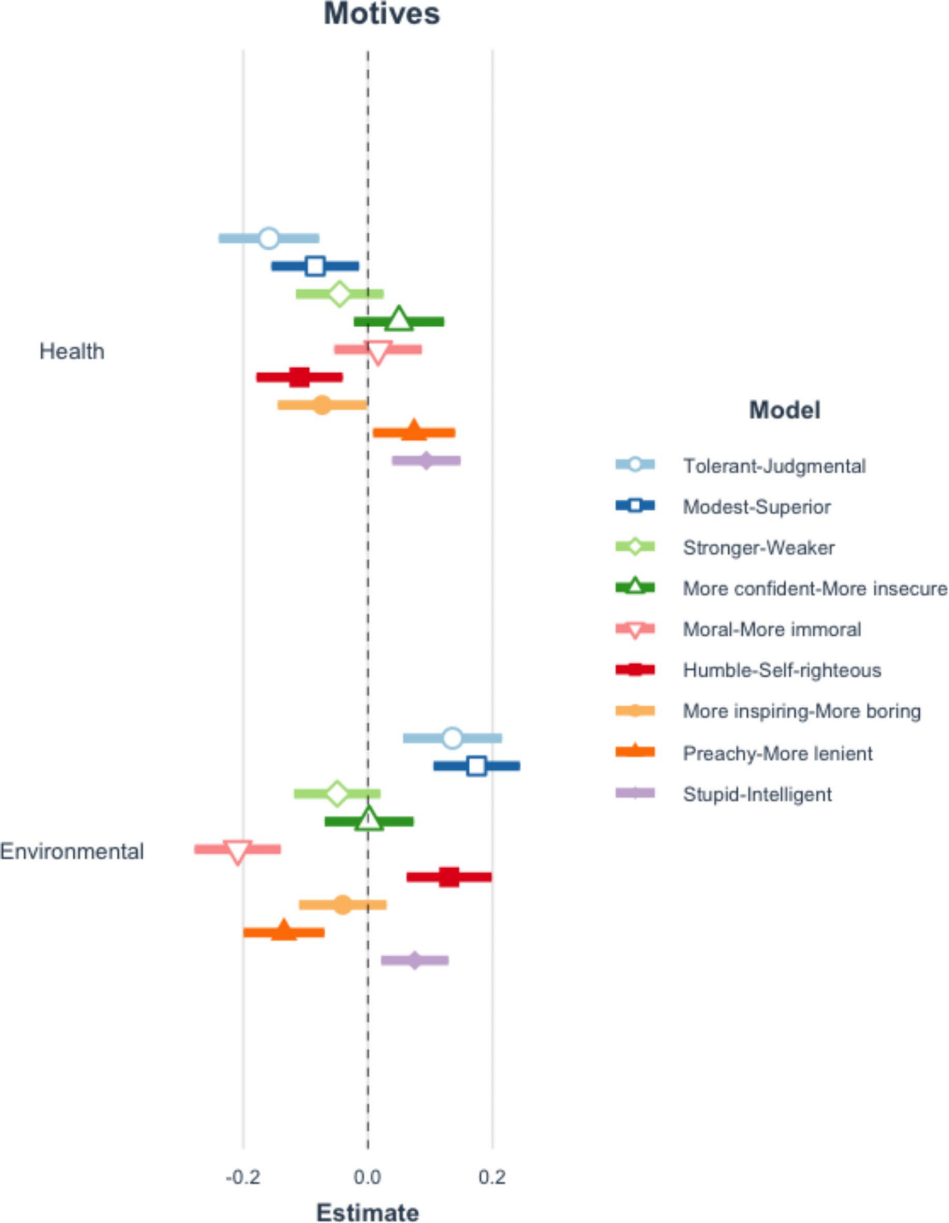
Coefficient plot of perceptions and motives mentioned. This figure reflects 9 separate multilevel models which examine the impact of mentioning either motive on the perceptions of the requester. Error bars represent 99% confidence intervals of estimates. Table A13 in the appendix presents the full results of all estimated models.

The vegetarian meal requester is perceived as less judgmental, self-righteous, and preachy, more modest (less superior), as well as more inspiring and intelligent when they mention health motives for their request compared to none. In contrast, they are perceived to be more judgmental, more self-righteous, more preachy, and less modest (more superior) when they mention environmental motives compared to none, however, they are also perceived to be more intelligent and more moral. See Fig. 4 and Appendix Table A13.

**Fig. 5:**
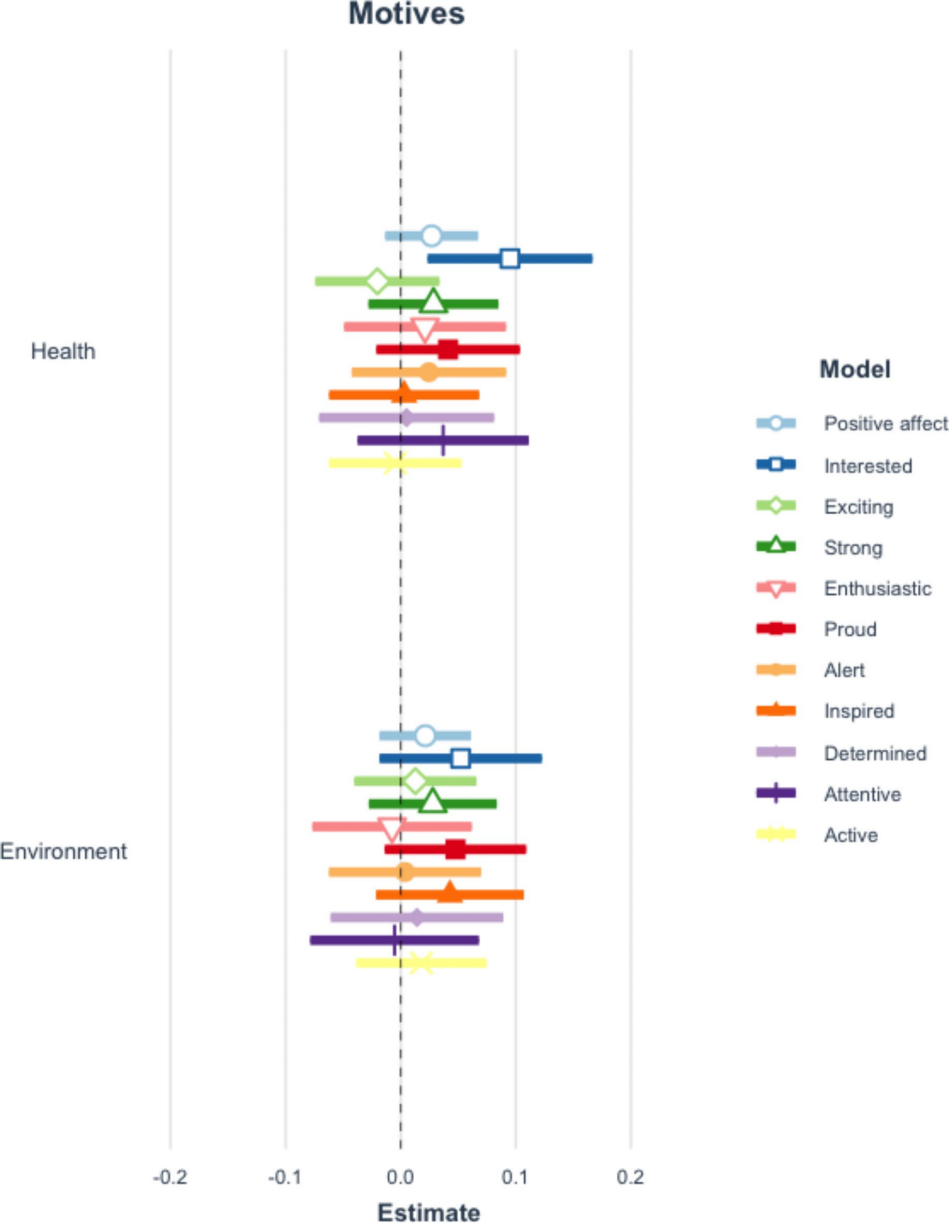
Coefficient plot of positive affect and motives mentioned. This figure reflects 11 separate multilevel models which examine the impact of mentioning either motive on positive affective responses. Error bars represent 99% confidence intervals of estimates. Table A14 in the appendix presents the full results of all estimated models.

There is no statistically significant relationship between positive affect overall and either motive being cited (health or environmental) when an individual requests a vegetarian meal option. Looking at specific emotions, however, we see that people are more interested when a person reports health reasons compared to when no reason is given. See Fig. 5 and Appendix Table A14.

**Fig. 6:**
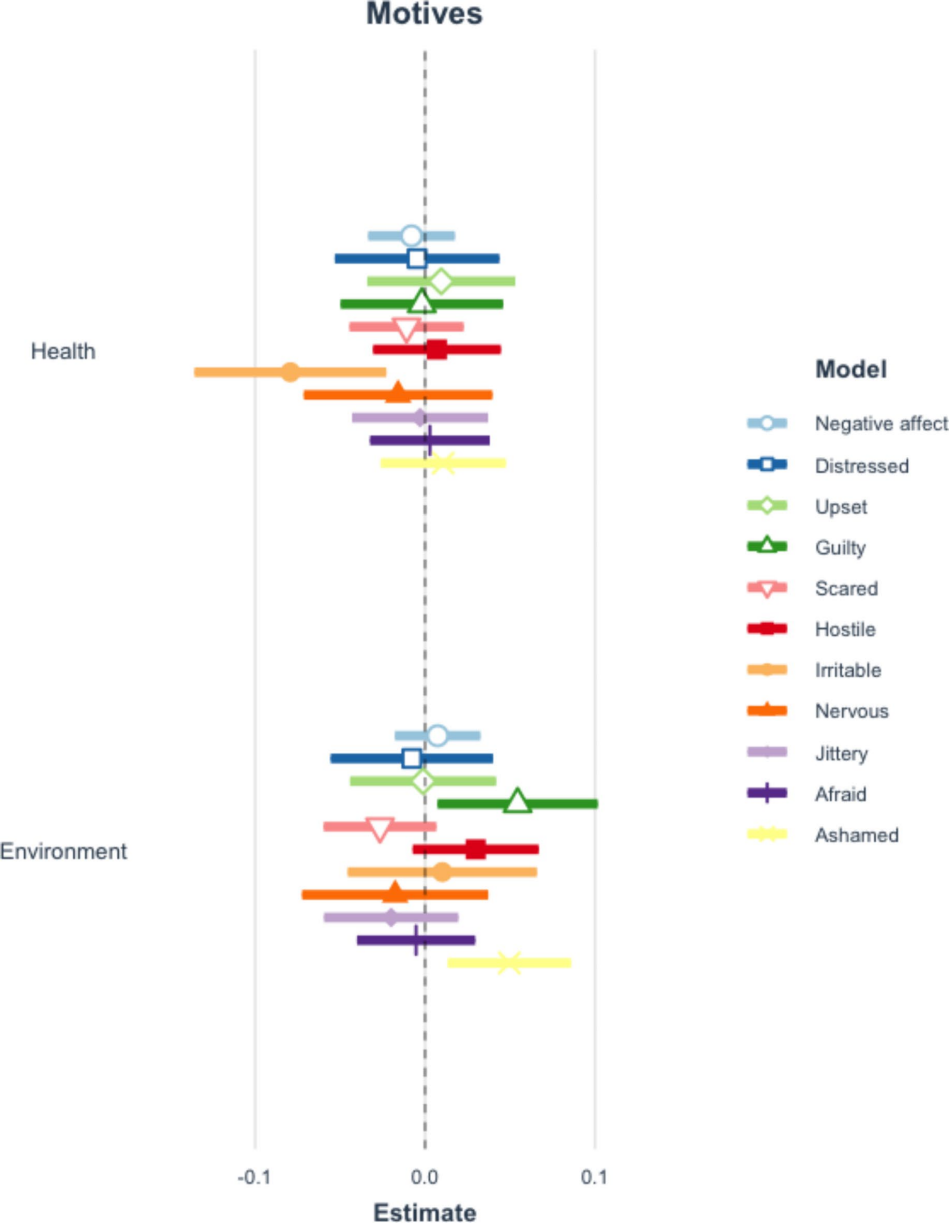
Coefficient plot of negative affect and motives mentioned. This figure reflects 11 separate multilevel models which examine the impact of mentioning either motive on negative affective responses. Error bars represent 99% confidence intervals of estimates. Table A15 in the appendix presents the full results of all estimated models.

There is also no statistically significant relationship between negative affect overall and mentioning either motive when requesting a vegetarian option. Looking at specific emotions, however, respondents report feeling less irritable when people give health reasons for refraining from meat consumption but more guilty and ashamed when they report environmental reasons, compared to when no reason is stated. See Fig. 6 and Appendix Table A15.

The evidence supports the hypothesis that those with environmental motives for their diet are perceived more negatively, while those with health motives are perceived more positively. The results on affective responses are less clear, with no statistically significant difference in overall affect by motive. Explaining that your diet is motivated by health elicits interest, while there are no significant positive emotional responses to environmental motives. In contrast, negative emotional responses do not arise when health reasons are cited, but meat eaters experience negative emotions when environmental reasons are mentioned.

### Meat attachment and responses

We conduct a multilevel analysis of respondents’ perceptions of and affective responses to vegetarian meal requests across four social meal settings to identify between-person variation in these variables across different levels of meat attachment. Our third pre-registered hypothesis (H3) was that meat eaters with high levels of meat attachment would respond less positively to vegetarian requests, in terms of both affect and perceptions of the meat abstainer, compared with those with relatively lower meat attachment. While we find no difference in affective responses, the evidence presented here on perceptions of the requestor strongly supports this hypothesis.

The respondents’ level of meat attachment is strongly predictive of their responses to the vegetarian meal requester. Firstly, meat attachment is positively associated with ratings of how judgmental, weak, insecure, immoral, self-righteous, boring, preachy, and stupid the requester is, as well as the perceived extent to which they feel superior. Second, meat attachment is negatively associated with positive affect overall and with all ten discrete positive emotions examined. Third, meat attachment is positively associated with negative affect overall. Higher levels of meat attachment are associated with higher levels of distress, upset, hostility, and irritability in response to the vegetarian meal request, however those with higher levels of meat attachment feel less guilty and ashamed. See Appendix Tables A16-18.

Turning to the question of whether the impact of mentioning a diet type or motive varies across different levels of meat attachment, we find no statistically significant impact of mentioning diet on either the perceptions of the requester or positive affective responses. Similarly, we find that affective responses to motives for diets when requesting a vegetarian meal do not vary across levels of meat attachment. However, perceptions of requestors when motives are explained do vary. Those with high levels of meat attachment tend to judge the requester as more moral and less self-righteous (more humble) when they mention health reasons compared to their less attached counterparts. In contrast, meat-attached respondents judge requesters to be more boring, less intelligent (more stupid), and less humble (more superior) when they are motivated by environmental reasons than those who are less attached to meat. See Appendix Table A19-24. Overall, while there are no significant differences between the affective responses of individuals across levels of meat attachment, the analysis strongly suggests that meat eaters who are very attached to meat perceive those with health motives for their diet more favourably than individuals with environmental motives.

### Contextual analysis

In an exploratory analysis, we examine whether the responses of interest vary across the four different meal scenarios. We find that meal requesters are perceived to be less preachy when making the request at a Pub or a BBQ, compared to when they are invited to a dinner in the respondent’s own home. When we examine the affective responses to the vegetarian meal request across the four settings there are many significant differences. Respondents report less positive affect overall when the request is made in a pub or restaurant compared to a dinner party at home. Similarly, all specific positive emotions except pride are lower in these settings than at home. Respondents report higher levels of pride on average in response to a request at a BBQ compared to a dinner party at home. Additionally, we find that respondents report less negative affect overall when the request comes in a pub or restaurant than when it relates to a dinner party at home. When we examine specific emotions, we find that in both the pub and restaurant settings respondents feel less distressed, scared, hostile, irritable, nervous, jittery and afraid, but more guilty, than they do hosting a dinner at home. Respondents also feel more ashamed on average in the restaurant compared to a dinner at home. There are also significant differences in emotional responses to the request for a vegetarian meal when hosting a BBQ compared to a dinner party. Meat eaters experience lower levels of negative affect overall and are less distressed, upset, scared, irritable, nervous, jittery, and afraid when the setting is a BBQ rather than a dinner.

There is an important distinction between the different meal scenarios that may be driving some of the different responses. In both the dinner party and the BBQ settings, respondents are hosting in their own homes and responsible for catering, whereas in the restaurant and pub scenarios, they are not. Agreeing to the meal request requires effort in the first set of scenarios and we might therefore expect more negative perceptions and stronger affective responses resulting from the request. We investigated if the outcomes of interest varied across the scenarios that involved catering and those that did not. We found that, on average, when the respondent was hosting the meal, they perceived the requester to be more judgmental, weaker, more insecure, and less moral than when the request was made in a setting where the meal was outside their home. Similarly, respondents had much stronger affective responses, both positive and negative, to requests for vegetarian meals when they were hosting. See Appendix Tables 25, 26, 27, 28, 29 and 30.

We also investigated if responses varied depending on whether requests came from a colleague, a friend, or a family member. Overall, there was little evidence that the nature of the relationship between the respondent and the requester mattered. The perceptions of the requester do not vary significantly depending on whether that person is a friend, colleague, or family member. However, we do find that the respondents reported feeling prouder on average when the requester was a family member compared to a colleague. See Appendix Tables 31, 32 and 33.

### Further exploratory analysis on additional outcome variables

In addition to perceptions and affective responses, we collected data on the respondent’s willingness to accommodate a vegetarian meal request in the two scenarios in the home (BBQ and Dinner party). People are generally supportive and accommodating of requests for meat-free meals; 82 per cent of people said they would happily provide a vegetarian option when hosting a BBQ, and 77 per cent when hosting a dinner party in their home. In both scenarios, fewer than 3 per cent of respondents said they would refuse the request. The willingness to accommodate a request was unimpacted by either diet or reason being mentioned.

We also examined the level of comfort that the respondents expected to feel while eating meat in the situation across all scenarios. The majority of people said that they would feel either extremely comfortable (42 per cent) or somewhat comfortable eating meat at the meal (32 per cent). We did not find their level of comfort to be impacted by any specific diet or reasons being mentioned. In projecting how comfortable they thought the requester would feel eating vegetarian food at this meal, most respondents thought that they would feel extremely comfortable (55 per cent) or somewhat comfortable (21 per cent). The expected comfort levels of the requester did not differ across diet types, however respondents thought that those citing environmental reasons would be more comfortable. See Appendix Table A34.

Finally, across all meal scenarios, most meat eaters were either very likely (59 per cent) or somewhat likely (20 per cent) to invite the individual to a similar occasion again in the future. Only a small percentage of individuals reported that they would be very unlikely to invite the person again in the future (3%). How likely individuals would be to invite this person again in future is not impacted by the mention of either diet type or motivation. See Appendix Table 35.

## Discussion

In a large pre-registered experimental vignette study, we examine responses to people requesting a vegetarian meal option across four everyday meal scenarios. More specifically we examine whether mentioning a diet (vegetarian or meat restrictor) and the motivation behind the request (health or environmental) impacts both people’s perceptions of the requester and their positive and negative affective responses to the request. The study provides rich insights into how a representative sample of British meat eaters report they would respond, on average, to a person making a vegetarian meal request. We also examine the differential effects of the setting in which the request is made (either in their own home at a dinner party or a BBQ or when sharing a meal at a pub or restaurant) as well as exploring the effects of the and the company a person is in.

The results indicate that mentioning diet type has little impact on perceptions of vegetarian meal requesters. When it comes to affective responses, however, we find that meat eaters report feeling more inspired and interested when the requester is a meat restrictor. These results are consistent with previous research indicating that meat eaters find those reducing meat consumption aspirational^27^, and that attitudes towards both diet types are generally positive^27,32^. The results may also be indicative of shifting social norms. Those abstaining from meat consumption in the UK, although still a minority, have increased to 15 per cent of the population, while flexitarians (those who restrict their meat consumption, eating it only occasionally) account for a further 14 per cent of the population^33^. Our results suggest that mentioning dietary lifestyle, irrespective of whether it involves restricting or avoiding meat altogether, does not lead to negative perceptions or affective responses. Meat restrictors may serve to inspire and interest others, potentially leading to even more widespread adoption of low or no-meat diets. Explaining your diet when requesting a vegetarian meal may also help to further shift dietary social norms.

In contrast, there is a good deal more evidence that mentioning motives affects perceptions of and affective responses to the requester. Overall, there is a general pattern of greater negative perceptions in response to environmental motivations (the requesters are thought to be more judgmental, more self-righteous, more preachy and more superior) and more positive responses (less judgmental, less self-righteous and preachy, less superior, more intelligent) when health motives are mentioned, compared to when no motive is stated. Importantly however, there is also evidence of some positive perceptions of those with environmental motives (they are seen as more intelligent and more moral), which may be explained by people having conflicted responses to the requester (e.g. they perceive them to be both judgmental and intelligent) or by different subgroups responding differently. Although there are no differences in overall positive or negative affect across motivations, there are some interesting differences in individual emotions experienced by respondents. Meat eaters feel interested and less irritated by those with health reasons for their diet, but guilty and ashamed when the requestor mentions environmental motives. This is suggestive of cognitive dissonance, or the ‘meat paradox’ whereby some people may want to eat meat but are aware of the environmental impact of doing so and do not want to contribute to climate change. Health reasons for not eating meat are more personal and do not infer a moral obligation on others to make similar choices.

Importantly, the level of meat attachment among meat eaters is highly predictive of both the perceptions of the requester and the affective responses to the request. The perceptions of how judgmental, superior, weak, insecure, immoral, self-righteous, boring, preachy and stupid the requester is, as well as all ten positive emotions and six out of ten negative emotions are associated with the meat eaters’ level of meat attachment. Meat attachment also moderates many of the impacts of mentioning motives. For example, the meat attached tend to respond more negatively towards those who report environmental motives, including viewing them as more boring, more stupid and more superior. In fact, the results suggest that those with low levels of meat attachment find those with environmental reasons for their diet to be more moral, more inspiring, and more intelligent. These findings highlight that not all meat eaters are alike in terms of their responses to people who refrain from or restrict their consumption of meat. When thinking about what these results imply for optimal communication strategies when requesting vegetarian meals it points to the old adage – ‘know your audience’.

A limitation of the study is that we only consider vegetarians and meat restrictors, we do not consider vegan diets. The number of treatment groups (7) when restricting the study to two diet types is quite large. Adding vegan as an additional diet type would have further increased the complexity of the analysis and required a much larger sample. Although more people across the Western world are shifting towards plant-based diets, particularly young adults, the numbers following a vegan diet in the UK are still quite small, at approximately 2 per cent^33^. Vegan diets are more restrictive than vegetarian diets as animal products such as eggs and dairy are not consumed. Vegans may therefore be perceived differently and requests for vegan options may elicit more negative responses than requests for vegetarian meals. Given the low carbon footprint and health benefits of vegan diets, exploring the social perceptions and responses to these diet types is important for future work.

Another limitation of this study is that we only consider health and environmental reasons for abstaining from meat. As outlined above in relation to restricting the diet types to two, adding additional motivations to the experiment would have required a substantially larger sample size and further increased the complexity of the analysis. Some studies combine motivations such as animal welfare and religion with environmental motivations and consider these jointly as ‘ethical’ or ‘moral’ motivations for abstaining from meat^34^. Although there is a big overlap between animal welfare and environmental motivations (many people are motivated by both), it is important to consider perceptions and responses to these explanations for diet type separately. Future studies could investigate whether those motivated by animal welfare are perceived differently, or if the emotional responses differ, compared with individuals with environmental reasons for their diet.

A final limitation of the study is the use of an experimental vignette design. Respondents are presented with four different social situations where they are eating with friends, colleagues, or family members. Both the scenarios and the individuals requesting the vegetarian meal are hypothetical. This abstraction detracts from the external validity of perceptions and affective responses of the respondents. Respondents may have a certain person or ‘type’ of person in mind or think of a person that is known to them already when providing answers. In a real-world scenario, responses to and perceptions of the requestor would likely be impacted by additional factors, such as their personality or likeability. It is not possible to eliminate these factors in our study but randomising the social contacts across treatment groups and meal scenarios goes some way to addressing this issue. Furthermore, for the ‘new colleague’ treatment scenarios, given that the colleague is new, respondents cannot have a person in mind when reporting their emotions and perceptions. Analysis of emotional responses and perceptions across the three different types of social contact did not find any statistically significant differences between them. This suggests that any preconceptions or connotations associated with the terms ‘friend’ and ‘family member’ did not overly influence the results. Future work could examine meat eaters’ responses in real meal scenarios to further shed light on how people respond to vegetarian meal requests made by people who are known to them.

We investigate the variation in responses across meal scenarios. Respondents reacted differently, and as expected, to the different scenarios presented in the vignettes. This implies that while the vignettes are not real-world scenarios, they are realistic scenarios and elicit situational responses from meat eaters. We find that on average responses are more negative to requests for vegetarian meals when the respondent is hosting the meal in their own home. Additionally, the most negative responses are when they are hosting a dinner party rather than a BBQ. These results are consistent with the differing levels of burden the request places on the host; in the public settings, there is no effort required on their part, while providing a vegetarian option in the informal setting of a BBQ is less onerous than in the more formal setting of a dinner.

Overall in this study, we find that perceptions of and affective responses towards meat restrictors are generally positive, and there are no negative perceptions of or responses to vegetarians. This suggests that there may be differences between how meat abstainers expect to be perceived and how they are actually perceived by meat eaters. Previous studies have shown that to escape negative reactions and stigma non-meat eaters often avoid expressing their preferences in social situations where they are in the minority. In a lab experiment, Bolderdijk and Cornelissen^24^ found vegetarians and vegans were worried about evoking stigma, and that these fears were not mitigated by giving a personal reason (e.g., health) for a meat-free diet rather than a moral reason (e.g., environment). Therefore, it is likely that meat abstainers expect any explanation of their diet will be perceived negatively. The results of this current work, as well as that of others^21,35^, indicate that differences do emerge in responses to meat-free diets depending on the underlying motives. An important next step would be to explore the extent of this potential perception gap between the responses vegetarian meal requesters expect compared to the average response meat eaters indicate they would have. If this gap was found to be extensive then communication strategies aimed at closing it could support vegetarians and meal restrictors to feel more comfortable requesting vegetarian meals and promote more sustainable dietary norms.

There is also evidence to suggest that campaigns focusing on motivations, i.e., the health or environmental benefits of reduced meat consumption, have differential effects across different groups. Those with no intention to reduce their meat intake are more likely to be persuaded by information on the environmental benefits, while those who already have intentions to reduce their meat consumption in the future are more likely to be prompted to do so by health information^36^. The results of this study offer some insights into these differential effects. It is possible that the negative perceptions and affective responses to those requesting vegetarian meals for environmental reasons could be partly explained by a lack of knowledge of the climate impact of meat production. Studies have shown that individuals across Europe tend to underestimate the environmental impact of meat consumption^37–39^. There is, therefore, a role for policymakers to inform consumers of the high carbon emissions produced by meat production. Campaigns focused on relaying this information may be effective across two channels. First, the information might encourage those without intentions to reduce meat consumption to do so. Second, increased awareness of the environmental benefits of abstaining from meat may reduce the negative perceptions of and responses towards those requesting vegetarian meals for environmental reasons. More social support for meat-free diets would help support those with intentions to reduce their meat consumption while also supporting those abstaining from meat to maintain their diets.

So when requesting vegetarian meals, is the optimal communication strategy to explain, or if you are explaining, are you losing? The answer is it depends. The results indicate that, on average, those wishing to abstain from meat should feel comfortable requesting vegetarian meals and mentioning their diet when doing so. In general, vegetarians should not fear a negative response from others, while meat restrictors may interest meat eaters and inspire them to make similar choices. When it comes to explaining motives however, those with environmental reasons for their diet face more negative reactions. In contrast, those with health reasons are more positively perceived, particularly among those with high levels of meat attachment. Mentioning the motive behind a vegetarian meal request can help or hinder depending on the motive and the company a person is in. In choosing if or when to explain motives, it is important to pick one’s battles.

## Methods

We carried out a pre-registered (https://aspredicted.org/94J_YBM*)* experimental vignette study to investigate the feelings and perceptions of meat eaters towards individuals who request a vegetarian meal. We used the online platform Prolific to conduct the study on a quasi-nationally representative sample of 1,222 UK adults (based on age, gender and ethnicity). Informed consent was obtained from all subjects. The respondents consist of 47 (3.9%) pescatarians (those who eat fish but not the flesh of other animals), 74 (6%) vegetarians (those who do not eat fish or meat), and 28 vegans (2.3%) (those who do not eat fish, meat, or any products derived from animals). There are 1,117 meat eaters in the sample – individuals that eat meat, with 161 (14.4%) reporting that they limit their consumption of meat. We restrict our analysis to the meat-eating subset of the overall representative sample. Of these 1,117 respondents, 1,050 provided complete demographic information. The remaining 67 answered ‘prefer not to say’ on one or more of the measures capturing age, gender, income and education level. See Appendix Table A3 for a breakdown of the sample’s characteristics.

The sample was determined using a two-sided two-sample t-test power calculation with an effect size of 0.2 and a significance level of 0.05/4 to account for multiple comparisons, as each respondent sees four meal scenarios, using R Studio 1.3 and the command power.t.test. The power was set to be 0.8. This resulted in a required minimum sample for each of the 7 groups (6 treatments and control) of 91 respondents. Our final sample size is considerably larger, increasing the power and allowing us to identify smaller effects.

Our experimental vignette methodology identifies within-person variation in perceptions and affective responses to vegetarian meal requestors across two variables, diet and motivation. The survey presented each respondent with four different meal scenarios where meat would usually be the default food served; a barbeque, a roast dinner, a pub carvery, and a Turkish restaurant. In the barbeque and dinner scenarios, the meat eater is hosting, and the requester asks if it would be possible for a vegetarian meal to be made available. In the public settings of the pub and restaurant, the requestor orders a vegetarian meal. These scenarios were chosen to capture the average perceptions of, and affective responses to, vegetarian meal requesters in typical social settings where eating meat is the norm. It might be the case however that these perceptions and responses differ across scenarios. For example, meat eaters might react more negatively to a vegetarian meal request when they are hosting compared to a request in a public setting. We, therefore, analyse the responses from all four meal scenarios together to determine the average impact of mentioning diet and/or motivation on perceptions and responses, and additionally analyse each meal scenario separately to identify any scenario-specific effects.

**Fig. 7:**
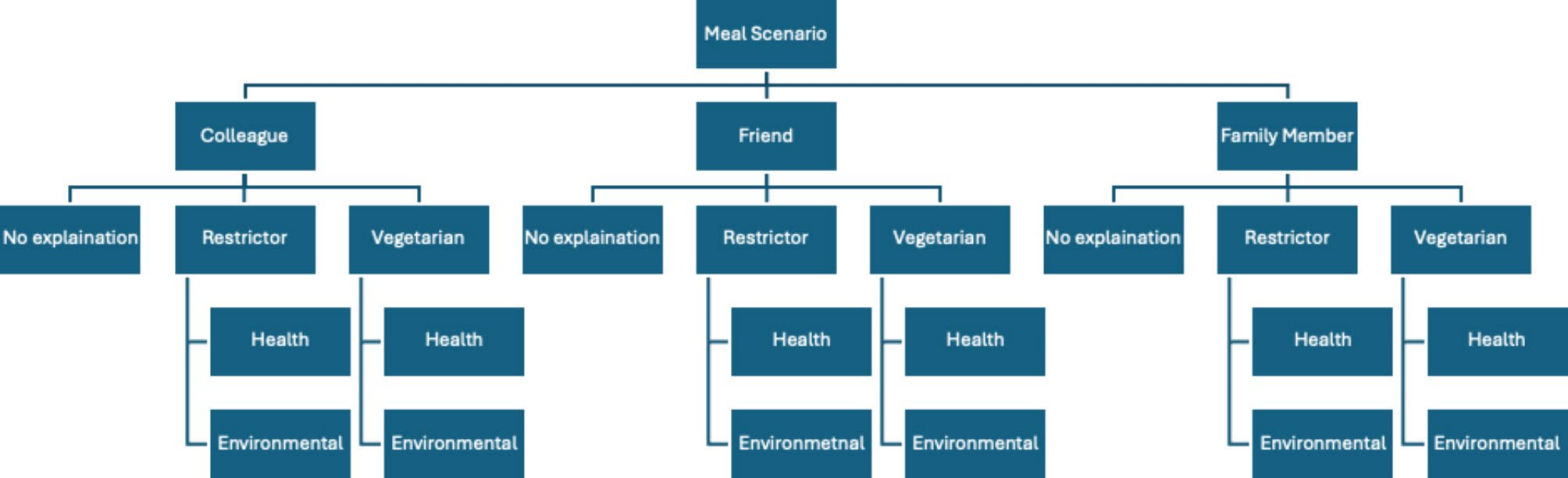
Randomisation process for each meal scenario. The process results in 7 vignettes for each of the three social groups, totalling 21 vignettes for each meal. Respondents see a total of 4 vignettes, one for each meal.

To determine whether the emotional responses and perceptions of meat eaters are impacted by requesters explaining either their diet or their diet and motivation, we initially designed text for seven different vignettes for each of the four meal scenarios. To ensure that any within individual variation in responses is explained by differences in the explanation or lack thereof for the meal choice alone, the experiment employed multiple randomisations finally resulting in 21 vignettes for each of the four meal scenarios. First, to remove any potential order effects, we randomised the order in which we presented the meal scenarios to respondents. Therefore, each respondent saw four different meal scenario vignettes (BBQ, Dinner, Pub, and Restaurant) but in a random order. Second, to ensure that the results were not driven by the social connection to the meal requestor, we randomised the social group that the respondent was eating with in each scenario (friends, family members, or colleagues). Figure 7 below illustrates the randomisation process for a meal scenario, producing 21 unique vignettes for each meal type. Each respondent saw four different vignettes, one of these 21 vignettes for each of the four meal types. After each meal scenario vignette, they answered questions on their affective responses to the request and perceptions of the requestor. This resulted in a total of 4,377 meal response observations Table 1 presents an example of the full text for four vignettes that a respondent might have seen.

**Table 1:** *Vignette examples*.

Example 1. Social situation –With family members at pub; Diet – Vegetarian; Motive – Environment	Imagine you are going out to a pub for a meal with a group of your family members. Having looked at the menu, you suggest that the group orders the carvery special that comes with a choice of one of three meat options and shared sides. All but one of your family members agree. They explain that they are now vegetarian for environmental reasons and order a separate vegetarian dish.
Example 2. Social situation – With colleague at Turkish restaurant; Diet – Meat restrictor; Motive – Health	*Imagine a new colleague has joined your team at work and you go out to dinner with them along with a group of other colleagues to a Turkish restaurant. Having looked at the menu*,* you suggest that the group orders the set tasting menu of meat based dishes that comes recommended and all but your new colleague agree. They explain that they are trying to restrict their meat consumption for health reasons and order a separate vegetarian dish.*
Example 3. Social situation – Invite colleague to a dinner party; Diet – Vegetarian; Motive – None	*Imagine a new colleague has joined your team at work and you invite them over to your house for a dinner party. You plan on providing the food and serving a roast dinner and let them know. They explain that they are a vegetarian and ask you if it would be possible for a vegetarian option to be made available.*
Example 4. Social situation – Invite friend to a BBQ; Diet – None; Motive – None	*Imagine you invite a friend over to your house for a barbecue. You let them know that you plan on providing the food and serving burgers and hotdogs. They ask you if it would be possible for a vegetarian option to be made available.*

In each vignette, the requestor (family member, friend, or colleague) either simply orders/requests a vegetarian meal, or orders and explains their diet (vegetarian or meat restrictor), or orders and explains both their diet and motivation (health or environmental reasons). The resulting control and treatment groups are presented in Table 2 below and the representation of the different scenarios in our dataset can be seen in Table 3. Our key variables of interest are diet type and motivation. Analysis of the results will determine if an individual meat eater’s perceptions of and affective responses to the vegetarian meal request are impacted by these factors. The study protocol was approved by the Ethics Review Board at The London School of Economics and Political Science. The study was carried out in accordance with this protocol and followed the school’s research ethics guidelines and regulations.

**Table 2:** Experimental vignette conditions.

	Diet	Motivation
Control	None	None
Treatment 1	Vegetarian	None
Treatment 2	Meat Restrictor	None
Treatment 3	Vegetarian	Environmental
Treatment 4	Meat Restrictor	Environmental
Treatment 5	Vegetarian	Health
Treatment 6	Meat Restrictor	Health

**Table 3:** Scenario representation in the dataset. This represents a total episode sample size of 4,377. The number of episodes involved in each model specification varies slightly due to non-responses for some outcomes.

	No motive	Health	Environment
No diet	614	0	0
Vegetarian	625	627	624
Meat restrictor	624	613	650

As our primary outcome variables, we asked respondents after each of the four scenario vignettes to report on their perceptions of the requester (measures adapted from Monin and colleagues^40^ by Weiper and Vonk^16^), and how the request would make them feel, in terms of a range of different measures of positive and negative affect measured using the PANAS scale^41^. The text presented to respondents to capture these variables is presented in Tables A1-A3 in the Appendix. As additional exploratory outcome variables, we also recorded their level of comfort eating meat in the meal scenario, the level of comfort they expected the requester to feel, and how likely they would be to invite the person to a similar occasion again. Additionally, in the case of the two home-based scenarios, we asked about their willingness to accommodate the request.

In the sample, the meat eaters’ perceptions of the vegetarian meal requesters are measured on a scale of 1–7, where 4 indicates neutral or no change in perceptions in response to the request for a meat-free meal. The values of 1 and 7 represent opposing traits, for example on the first scale presented 1 = more tolerant and 7 = more judgemental. In this example, values of 1–3 indicate that the requester is perceived to be tolerant, lower numbers indicating increasingly more tolerant. Conversely, values of 5–7 indicate the requester is perceived to be judgemental, with higher values indicating increasingly more judgemental. See Appendix Fig. 1 for an example of the presentation of these questions and A3 and A6 for a full list of opposing traits and summary statistics respectively. The distance from the midpoint capturing changes in perceptions) ranges from the highest average value of 0.562 (on a scale of 1–3) for more moral (SD = 0.966) to the lowest average value of 0.071 for more judgemental (SD = 1.002). Emotional responses (both positive and negative affect) are measured by asking respondents to what extent the request for a vegetarian meal makes them feel a particular emotion, on a scale of 1–5, where 1 indicates ‘not at all’ and 5 indicates ‘extremely’. The average level of positive affect is low at 1.795 (SD = 0.787), ranging from the lowest average score of 1.52 (0.897) for feeling strong to the highest score of 2.32 (SD = 1.072) for feeling interested. By comparison, the overall level of negative affect reported is even lower, with an average negative affect rating of 1.224 (SD = 0.376) and ranging from an average score of 1.111 (SD = 0.399) for feeling scared to 1.4 (SD = 0.745) for feeling irritable on a scale of 1–5. See Appendix Table A7 for further details.

We also estimated the level of meat attachment for each respondent, using a scale that captures the extent to which a person has a positive bond with meat consumption across four different dimensions^31^: hedonic, affinity, dependence, and entitlement. Respondents indicated the extent to which they agree with each of the fifteen statements and their answers are combined to measure overall meat attachment. See Appendix A4 for a full list of statements in each category.

The average level of meat attachment among the sample is 3.457 (SD = 0.871), averaging over 15 statements presented that assess an aspect of meat attachment on a scale of 1–5 where 1 indicates the respondent strongly disagrees with the statement and 5 indicates they strongly agree. This scale can be broken down into subcomponents of meat attachment^31^. The average sample scores for these subcomponents are as follows: Affinity (mean = 3.986, SD = 0.875), hedonic (mean, 3.685, SD = 0.956), entitlement (mean = 3.272, SD = 0.971) and dependence (mean = 3.008, SD = 1.072). See Appendix Table A8.

When we look at the differences in meat attachment across age groups we see that the over 65s are significantly more meat-attached (mean = 3.56, SD = 0.8) than the under 25s (mean = 3.34, SD = 0.92), men have higher meat attachment (mean = 3.63, SD = 0.772) than women (mean = 3.29, SD = 0.822) and those with no higher education have greater meat attachment (mean = 3.53, SD = 0.839) than those without (mean = 3.4, SD = 0.795). There are no significant differences in meat attachment across income groups. See Appendix Table A9.

In the main analysis, we examine the responses of the meat eaters to the vegetarian meal request. Given that each respondent provided multiple meal responses, we use multilevel modelling to account for the nested structure of the data with a random intercept term at the individual level using the lme4 package in R Studio 1.3^42^. Models were fit using maximum likelihood estimation. In the work’s primary specification, we examine if and how the perceptions of requesters and the affective responses to the request vary depending on whether and which diets and or motivations are stated. In further exploratory analysis, we include interaction terms between these treatment variables of interest and the level of meat attachment of the respondent. In all cases, we applied Benjamini-Hochberg adjustments to all estimates based on within table groupings^43^. These adjustments mitigate the potential for the occurrence of false positives arising from the investigation of multiple outcomes simultaneously by adjusting the p-values associated with each estimate based on their rank within the p-values for a family of models. Although our pre-registration did not include correction for multiple testing, we implemented the Benjamini-Hochberg procedure to ensure the robustness of our results following reviewer comments prior to publication.

## Electronic supplementary material

Below is the link to the electronic supplementary material.

Supplementary Material 1

## Data Availability

The datasets used and analysed during the current study are available from the corresponding author upon reasonable request. Please contact Dr Kate Laffan at k.m.laffan@lse.ac.uk.
