# Supplementary material for: The impact of explaining vegetarian meal requests on the affective responses and perceptions of meat eaters
Source: Sci Rep. 2024 Oct 16;14:24262. doi: 10.1038/s41598-024-74479-1 (PMC11484982; doi:10.1038/s41598-024-74479-1)
Supplement: Supplementary file 1 — Supplementary Material 1 [file 41598_2024_74479_MOESM1_ESM.docx]

**Appendix**

**PART I. Background details and descriptive statistics**

**Table A1**

*Positive affect questions*

| To what extent would this person's order make you feel the following emotions? | | | | | |
| --- | --- | --- | --- | --- | --- |
|  | Not at all | A little | Somewhat | Very | Extremely |
| Interested | O | O | O | O | O |
| Distressed | O | O | O | O | O |
| Excited | O | O | O | O | O |
| Upset | O | O | O | O | O |
| Strong | O | O | O | O | O |
| Guilty | O | O | O | O | O |
| Scared | O | O | O | O | O |
| Hostile | O | O | O | O | O |
| Enthusiastic | O | O | O | O | O |
| Proud | O | O | O | O | O |

*Note.* The exact wording applies to the Pub and Restaurant scenarios. In the text for the BBQ and dinner

scenarios the word ‘order’ is replaced by ‘request’.

**Table A2**

*Negative affect questions*

| To what extent would this person's order make you feel the following emotions? | | | | | |
| --- | --- | --- | --- | --- | --- |
|  | Not at all | A little | Somewhat | Very | Extremely |
| Interested | O | O | O | O | O |
| Distressed | O | O | O | O | O |
| Excited | O | O | O | O | O |
| Upset | O | O | O | O | O |
| Strong | O | O | O | O | O |
| Guilty | O | O | O | O | O |
| Scared | O | O | O | O | O |
| Hostile | O | O | O | O | O |
| Enthusiastic | O | O | O | O | O |
| Proud | O | O | O | O | O |

*Note.* The exact wording applies to the Pub and Restaurant scenarios. In the text for the BBQ and dinner

scenarios the word ‘order’ is replaced by ‘request’.

**Figure A1**

*Perception question example*

| Based on their order, on a scale of 1 to 7, where 1 is 'more tolerant' and 7 is 'more judgmental', how would you now perceive this person? | | |
| --- | --- | --- |
| More tolerant | No different | More judgemental |

| 1 | 2 | 3 | 4 | 5 | 6 | 7 |
| --- | --- | --- | --- | --- | --- | --- |

| 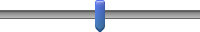 |
| --- |

**Table A3**

*Perception questions*

| Based on their order, on a scale of 1 to 7, where 1 is 'more tolerant' and 7 is 'more judgmental', how would you now perceive this person?  Based on their order, on a scale of 1 to 7, where 1 is 'more modest' and 7 is 'feels more superior', how would you now perceive this person?  Based on their order, on a scale of 1 to 7, where 1 is 'stronger' and 7 is 'weaker', how would you now perceive this person?  Based on their order, on a scale of 1 to 7, where 1 is 'more confident' and 7 is 'more insecure', how would you now perceive this person?  Based on their order, on a scale of 1 to 7, where 1 is 'more moral' and 7 is 'more immoral', how would you now perceive this person?  Based on their order, on a scale of 1 to 7, where 1 is 'more humble' and 7 is 'more self-righteous', how would you now perceive this person?  Based on their order, on a scale of 1 to 7, where 1 is 'more inspiring' and 7 is 'more boring', how would you now perceive this person?  Based on their order, on a scale of 1 to 7, where 1 is 'more preachy' and 7 is 'more lenient', how would you now perceive this person?  Based on their order, on a scale of 1 to 7, where 1 is 'more stupid' and 7 is 'more intelligent', how would you now perceive this person? |
| --- |

*Note.* The exact wording applies to the Pub and Restaurant scenarios. In the text for the BBQ and dinner

scenarios the word ‘order’ is replaced by ‘request’.

**Table A4**

*Meat Attachment Statements*

| Hedonic |
| --- |
| To eat meat is one of the good pleasures in life. |
| I love meals with meat. |
| A good steak is without comparison. |
| Affinity |
| I feel bad when I think of eating meat. |
| To eat meat is disrespectful towards life and the environment. |
| Meat reminds me of diseases. |
| By eating meat I’m reminded of the death and suffering of animals. |
| Dependence |
| Meat is irreplaceable in my diet. |
| I would feel fine with a meatless diet. |
| If I couldn’t eat meat I would feel weak. |
| I don’t picture myself without eating meat regularly. |
| If I was forced to stop eating meat I would feel sad. |
| Entitlement |
| According to our position in the food chain, we have the right to eat meat. |
| To eat meat is an unquestionable right of every person. |
| Eating meat is a natural and undisputable practice. |

**Table A5**

*Descriptive statistics for sample*

|  | Proportion |
| --- | --- |
| Age |  |
| 18-24 | 0.076 |
| 25-34 | 0.216 |
| 35-44 | 0.189 |
| 45-54 | 0.172 |
| 55-64 | 0.218 |
| 65+ | 0.128 |
| Gender |  |
| Male | 0.510 |
| Female | 0.488 |
| Neither | 0.002 |
| Income |  |
| Less than £20,000 | 0.171 |
| £20,000- £39,999 | 0.376 |
| £40,000-£59,999 | 0.251 |
| £60,000-£99,999 | 0.159 |
| More than £100,000 | 0.043 |
| Education |  |
| No higher education | 0.423 |
| Higher education | 0.577 |

*Note.* n=1,050

**Table A6**

*Descriptive statistics for perceptions*

|  | Mean (Neutral = 4) | Distance from Midpoint | SD |
| --- | --- | --- | --- |
| Perceptions |  |  |  |
| Tolerant - Judgmental | 4.071 | 0.071 | 1.002 |
| Modest - Feels superior | 4.175 | 0.175 | 0.874 |
| Stronger - Weaker | 3.667 | 0.333 | 0.924 |
| Confident - Insecure | 3.480 | 0.520 | 1.001 |
| Moral - Immoral | 3.438 | 0.562 | 0.966 |
| Humble - Self- righteous | 4.221 | 0.221 | 0.940 |
| Inspiring - Boring | 3.814 | 0.186 | 1.008 |
| Preachy - More lenient | 3.712 | 0.288 | 0.861 |
| Stupid - Intelligent | 4.204 | 0.204 | 0.774 |

*Note.* n = 4,377

**Table A7**

*Descriptive statistics for affect*

|  | Mean | SD |
| --- | --- | --- |
| Negative affect | 1.224 | 0.376 |
| Distressed | 1.239 | 0.592 |
| Upset | 1.204 | 0.556 |
| Guilty | 1.323 | 0.645 |
| Scared | 1.111 | 0.399 |
| Hostile | 1.153 | 0.483 |
| Irritable | 1.403 | 0.745 |
| Ashamed | 1.183 | 0.496 |
| Nervous | 1.315 | 0.659 |
| Jittery | 1.188 | 0.527 |
| Afraid | 1.125 | 0.429 |
| Positive affect | 1.795 | 0.787 |
| Interested | 2.322 | 1.072 |
| Excited | 1.526 | 0.882 |
| Strong | 1.519 | 0.897 |
| Enthusiastic | 1.846 | 1.070 |
| Proud | 1.651 | 0.999 |
| Alert | 1.802 | 0.976 |
| Inspired | 1.819 | 1.027 |
| Determined | 1.728 | 1.029 |
| Attentive | 2.100 | 1.106 |
| Active | 1.638 | 0.945 |
| Comfort you | 1.94 | 1.037 |
| Comfort them | 1.838 | 1.103 |
| Invite back | 1.739 | 1.07 |

*Note.* n = 4,377

**Table A8**

*Descriptive statistics for meat attachment*

|  | Mean | SD |
| --- | --- | --- |
| Meat attachment overall | 3.457 | 0.817 |
| Meat attachment (affinity) | 3.986 | 0.875 |
| Meat attachment (hedonic) | 3.685 | 0.956 |
| Meat attachment (dependence) | 3.008 | 1.072 |
| Meat attachment (entitlement) | 3.272 | 0.971 |
| Meat attachment by age |  |  |
| 18-24 | 3.34 | 0.92 |
| 25-34 | 3.46 | 0.805 |
| 35-44 | 3.51 | 0.771 |
| 45-54 | 3.47 | 0.794 |
| 55-64 | 3.38 | 0.851 |
| 65+ | 3.56 | 0.8 |
| Meat attachment by gender |  |  |
| Male | 3.63 | 0.772 |
| Female | 3.29 | 0.822 |
| Neither | 2.47 | 0.471 |
| Meat attachment by income |  |  |
| Less than £20,000 | 3.45 | 0.88 |
| £20,000- £39,999 | 3.46 | 0.807 |
| £40,000-£59,999 | 3.43 | 0.824 |
| £60,000-£99,999 | 3.53 | 0.749 |
| More than £100,000 | 3.56 | 0.751 |
| Meat attachment by higher education |  |  |
| No higher education | 3.53 | 0.839 |
| Higher education | 3.4 | 0.795 |

*Note.* n for meat attachment statistics= 1,117; n for meat attachment by demographics= 1,050

**Table A9**

*Regression analysis of overall meat attachment and demographics*

|  | |
| --- | --- |
|  | Dependent variable: |
|  |  |
|  | Meat attachment |
|  | |
| Reference: Under 25 |  |
| 25-34 | 0.095 |
|  | (0.106) |
| 35-44 | 0.139 |
|  | (0.107) |
| 45-54 | 0.093 |
|  | (0.108) |
| 55-64 | 0.022 |
|  | (0.104) |
| 65+ | 0.244** |
|  | (0.114) |
| Reference: Male |  |
| Female | -0.335*** |
|  | (0.049) |
| Neither | -1.115** |
|  | (0.563) |
|  |  |
| Reference: No higher education |  |
| Higher education | -0.158*** |
|  | (0.051) |
| Reference: Under £20,000 |  |
| £20,000-£39,999 | 0.006 |
|  | (0.072) |
| £40,000-£59,999 | -0.026 |
|  | (0.078) |
| £60,000-£99,999 | 0.104 |
|  | (0.088) |
| Over £100,000 | 0.156 |
|  | (0.135) |
|  |  |
| Constant | 3.608*** |
|  | (0.104) |
| Observations | 1,050 |
| R2 | 0.066 |
| Adjusted R2 | 0.055 |
| Residual Std. Error | 0.790 (df = 1037) |
| F Statistic | 6.079*** (df = 12; 1037) |
|  | |
| *Note.**p<0.1**p<0.05***p<0.01 | |

**PART II. Main Analyses**

**Table A10**

*Perceptions and diet type*

|  | Dependent variable: | | | | | | | | |
| --- | --- | --- | --- | --- | --- | --- | --- | --- | --- |
|  |  | | | | | | | | |
|  | Tolerant  –  Judgmental | Modest  –  Feels superior | Stronger  –  Weaker | Confident  –  Insecure | Moral  –  Immoral | Humble  –  Self-righteous | Inspiring  –  Boring | Preachy  –  More lenient | Stupid  –  Intelligent |
|  | (1) | (2) | (3) | (4) | (5) | (6) | (7) | (8) | (9) |
|  | | | | | | | | | |
| Vegetarian | 0.0004 | -0.013 | -0.065^*^ | 0.030 | -0.022 | -0.032 | -0.091^***^ | -0.024 | 0.050^*^ |
|  | (0.040) | (0.035) | (0.035) | (0.036) | (0.035) | (0.034) | (0.035) | (0.033) | (0.027) |
|  |  |  |  |  |  |  |  |  |  |
| Meat restrictor | 0.042 | 0.040 | -0.071^**^ | 0.042 | -0.041 | -0.004 | -0.070^**^ | -0.050 | 0.050^*^ |
|  | (0.040) | (0.035) | (0.034) | (0.035) | (0.035) | (0.034) | (0.035) | (0.032) | (0.027) |
|  |  |  |  |  |  |  |  |  |  |
| Constant | 4.052^***^ | 4.163^***^ | 3.722^***^ | 3.447^***^ | 3.466^***^ | 4.236^***^ | 3.881^***^ | 3.743^***^ | 4.160^***^ |
|  | (0.039) | (0.034) | (0.035) | (0.038) | (0.036) | (0.036) | (0.038) | (0.033) | (0.029) |
|  |  |  |  |  |  |  |  |  |  |
|  | | | | | | | | | |
| Observations | 4,362 | 4,362 | 4,362 | 4,362 | 4,362 | 4,362 | 4,362 | 4,362 | 4,362 |
| Log Likelihood | -5,794.174 | -5,203.809 | -5,258.532 | -5,461.330 | -5,347.142 | -5,262.037 | -5,420.906 | -4,979.211 | -4,281.487 |
| Akaike Inf. Crit. | 11,598.350 | 10,417.620 | 10,527.060 | 10,932.660 | 10,704.280 | 10,534.070 | 10,851.810 | 9,968.423 | 8,572.975 |
| Bayesian Inf. Crit. | 11,630.250 | 10,449.520 | 10,558.970 | 10,964.560 | 10,736.190 | 10,565.980 | 10,883.720 | 10,000.330 | 8,604.878 |
| Note.^*^p<0.1, ^**^p<0.05, ^***^p<0.01; Coefficients significant according to Benjamini-Hochberg threshold in bold. | | | | | | | | | |

**Table A11**

*Positive affect and diet type*

|  | Dependent variable: | | | | | | | | | | |
| --- | --- | --- | --- | --- | --- | --- | --- | --- | --- | --- | --- |
|  |  | | | | | | | | | | |
|  | Positive Affect | Interested | Excited | Strong | Enthusiastic | Proud | Alert | Inspired | Determined | Attentive | Active |
|  | (1) | (2) | (3) | (4) | (5) | (6) | (7) | (8) | (9) | (10) | (11) |
|  | | | | | | | | | | | |
| Vegetarian | 0.036^*^ | 0.083^**^ | -0.022 | 0.034 | 0.030 | 0.066^**^ | 0.038 | 0.032 | 0.005 | 0.054 | 0.034 |
|  | (0.020) | (0.035) | (0.026) | (0.028) | (0.035) | (0.031) | (0.033) | (0.032) | (0.037) | (0.036) | (0.028) |
|  |  |  |  |  |  |  |  |  |  |  |  |
| Meat restrictor | 0.030 | **0.107^***^** | -0.037 | 0.018 | 0.029 | 0.042 | 0.016 | **0.100^***^** | 0.017 | 0.0001 | 0.027 |
|  | (0.020) | (0.035) | (0.026) | (0.028) | (0.034) | (0.031) | (0.033) | (0.032) | (0.037) | (0.036) | (0.028) |
|  |  |  |  |  |  |  |  |  |  |  |  |
| Constant | 1.766^***^ | 2.241^***^ | 1.551^***^ | 1.498^***^ | 1.821^***^ | 1.606^***^ | 1.778^***^ | 1.762^***^ | 1.717^***^ | 2.077^***^ | 1.609^***^ |
|  | (0.027) | (0.039) | (0.031) | (0.032) | (0.039) | (0.036) | (0.036) | (0.037) | (0.039) | (0.040) | (0.034) |
|  |  |  |  |  |  |  |  |  |  |  |  |
|  | | | | | | | | | | | |
| Observations | 4,364 | 4,368 | 4,368 | 4,368 | 4,368 | 4,368 | 4,364 | 4,364 | 4,364 | 4,364 | 4,364 |
| Log Likelihood | -3,366.478 | -5,531.209 | -4,407.789 | -4,575.880 | -5,472.330 | -5,019.305 | -5,197.112 | -5,176.539 | -5,643.239 | -5,679.398 | -4,693.281 |
| Akaike Inf. Crit. | 6,742.955 | 11,072.420 | 8,825.577 | 9,161.760 | 10,954.660 | 10,048.610 | 10,404.220 | 10,363.080 | 11,296.480 | 11,368.800 | 9,396.562 |
| Bayesian Inf. Crit. | 6,774.861 | 11,104.330 | 8,857.488 | 9,193.671 | 10,986.570 | 10,080.520 | 10,436.130 | 10,394.980 | 11,328.380 | 11,400.700 | 9,428.468 |
| Note.^*^p<0.1, ^**^p<0.05, ^***^p<0.01; Coefficients significant according to Benjamini-Hochberg threshold in bold. | | | | | | | | | | | |

**Table A12**

*Negative affect and diet type*

|  | Dependent variable: | | | | | | | | | | |
| --- | --- | --- | --- | --- | --- | --- | --- | --- | --- | --- | --- |
|  |  | | | | | | | | | | |
|  | Negative Affect | Distressed | Upset | Guilty | Scared | Hostile | Irritable | Ashamed | Nervous | Jittery | Afraid |
|  | (1) | (2) | (3) | (4) | (5) | (6) | (7) | (8) | (9) | (10) | (11) |
|  | | | | | | | | | | | |
| Vegetarian | 0.014 | 0.010 | 0.027 | 0.029 | -0.003 | 0.028 | -0.003 | 0.012 | -0.018 | 0.004 | 0.045^***^ |
|  | (0.013) | (0.024) | (0.021) | (0.024) | (0.017) | (0.018) | (0.028) | (0.018) | (0.027) | (0.020) | (0.017) |
|  |  |  |  |  |  |  |  |  |  |  |  |
| Meat restrictor | 0.014 | -0.012 | 0.036^*^ | 0.040^*^ | -0.013 | 0.042^**^ | 0.027 | 0.022 | -0.033 | -0.004 | 0.021 |
|  | (0.012) | (0.024) | (0.021) | (0.023) | (0.016) | (0.018) | (0.028) | (0.018) | (0.027) | (0.020) | (0.017) |
|  |  |  |  |  |  |  |  |  |  |  |  |
| Constant | 1.212^***^ | 1.240^***^ | 1.176^***^ | 1.296^***^ | 1.118^***^ | 1.123^***^ | 1.390^***^ | 1.169^***^ | 1.336^***^ | 1.188^***^ | 1.097^***^ |
|  | (0.014) | (0.023) | (0.021) | (0.024) | (0.016) | (0.019) | (0.028) | (0.019) | (0.026) | (0.020) | (0.017) |
|  |  |  |  |  |  |  |  |  |  |  |  |
|  | | | | | | | | | | | |
| Observations | 4,364 | 4,368 | 4,368 | 4,368 | 4,368 | 4,368 | 4,364 | 4,364 | 4,364 | 4,364 | 4,364 |
| Log Likelihood | -996.371 | -3,525.084 | -3,130.768 | -3,626.832 | -1,883.751 | -2,494.988 | -4,310.887 | -2,476.928 | -4,068.140 | -2,803.444 | -2,125.417 |
| Akaike Inf. Crit. | 2,002.743 | 7,060.168 | 6,271.535 | 7,263.665 | 3,777.502 | 4,999.976 | 8,631.773 | 4,963.856 | 8,146.281 | 5,616.888 | 4,260.834 |
| Bayesian Inf. Crit. | 2,034.648 | 7,092.079 | 6,303.445 | 7,295.575 | 3,809.413 | 5,031.886 | 8,663.679 | 4,995.762 | 8,178.186 | 5,648.793 | 4,292.739 |
|  | | | | | | | | | | | |
| Note.^*^p<0.1, ^**^p<0.05, ^***^p<0.01; Coefficients significant according to Benjamini-Hochberg threshold in bold. | | | | | | | | | | | |

**Table A13**

*Motives and perceptions*

|  | | | | | | | | | |
| --- | --- | --- | --- | --- | --- | --- | --- | --- | --- |
|  | Dependent variable: | | | | | | | | |
|  |  | | | | | | | | |
|  | Tolerant  –  Judgemental | Modest  –  Feels superior | Stronger  –  Weaker | Confident  –  Insecure | Moral  –  Immoral | Humble  –  Self-righteous | Inspiring  –  Boring | Preachy  –  More lenient | Stupid  –  Intelligent |
|  | (1) | (2) | (3) | (4) | (5) | (6) | (7) | (8) | (9) |
|  | | | | | | | | | |
| Health motives | **-0.159^***^** | **-0.084^***^** | -0.045^*^ | 0.050^*^ | 0.016 | **-0.110^***^** | **-0.073^***^** | **0.074^***^** | **0.094^***^** |
|  | (0.031) | (0.027) | (0.027) | (0.028) | (0.027) | (0.027) | (0.028) | (0.026) | (0.021) |
|  |  |  |  |  |  |  |  |  |  |
| Environmental motives | **0.136^***^** | **0.175^***^** | -0.049^*^ | 0.002 | **-0.209^***^** | **0.131^***^** | -0.040 | **-0.135^***^** | **0.075^***^** |
|  | (0.031) | (0.027) | (0.027) | (0.028) | (0.027) | (0.026) | (0.027) | (0.025) | (0.021) |
|  |  |  |  |  |  |  |  |  |  |
| Constant | 4.077^***^ | 4.148^***^ | 3.691^***^ | 3.463^***^ | 3.495^***^ | 4.214^***^ | 3.845^***^ | 3.729^***^ | 4.154^***^ |
|  | (0.027) | (0.023) | (0.025) | (0.028) | (0.027) | (0.026) | (0.028) | (0.023) | (0.022) |
|  |  |  |  |  |  |  |  |  |  |
|  | | | | | | | | | |
| Observations | 4,362 | 4,362 | 4,362 | 4,362 | 4,362 | 4,362 | 4,362 | 4,362 | 4,362 |
| Log Likelihood | -5,758.325 | -5,166.499 | -5,258.612 | -5,460.223 | -5,309.887 | -5,229.247 | -5,420.650 | -4,951.362 | -4,271.744 |
| Akaike Inf. Crit. | 11,526.650 | 10,343.000 | 10,527.230 | 10,930.450 | 10,629.770 | 10,468.490 | 10,851.300 | 9,912.724 | 8,553.488 |
| Bayesian Inf. Crit. | 11,558.550 | 10,374.900 | 10,559.130 | 10,962.350 | 10,661.680 | 10,500.400 | 10,883.200 | 9,944.628 | 8,585.391 |
|  | | | | | | | | | |
| Note.^*^p<0.1, ^**^p<0.05, ^***^p<0.01; Coefficients significant according to Benjamini-Hochberg threshold in bold. | | | | | | | | | |

**Table A14**

*Motives and positive affect*

|  | Dependent variable: | | | | | | | | | | |
| --- | --- | --- | --- | --- | --- | --- | --- | --- | --- | --- | --- |
|  |  | | | | | | | | | | |
|  | Positive Affect | Interested | Excited | Strong | Enthusiastic | Proud | Alert | Inspired | Determined | Attentive | Active |
|  | (1) | (2) | (3) | (4) | (5) | (6) | (7) | (8) | (9) | (10) | (11) |
|  | | | | | | | | | | | |
| Health motives | 0.027^*^ | **0.095^***^** | -0.020 | 0.028 | 0.021 | 0.041^*^ | 0.024 | 0.003 | 0.005 | 0.037 | -0.005 |
|  | (0.016) | (0.028) | (0.021) | (0.022) | (0.027) | (0.024) | (0.026) | (0.025) | (0.030) | (0.029) | (0.022) |
|  |  |  |  |  |  |  |  |  |  |  |  |
| Environmental motives | 0.021 | 0.052^*^ | 0.013 | 0.028 | -0.008 | 0.048^**^ | 0.004 | 0.043^*^ | 0.014 | -0.005 | 0.018 |
|  | (0.015) | (0.027) | (0.021) | (0.022) | (0.027) | (0.024) | (0.026) | (0.025) | (0.029) | (0.028) | (0.022) |
|  |  |  |  |  |  |  |  |  |  |  |  |
| Constant | 1.781^***^ | 2.281^***^ | 1.527^***^ | 1.504^***^ | 1.842^***^ | 1.627^***^ | 1.793^***^ | 1.806^***^ | 1.721^***^ | 2.091^***^ | 1.632^***^ |
|  | (0.023) | (0.030) | (0.025) | (0.025) | (0.030) | (0.028) | (0.027) | (0.029) | (0.028) | (0.031) | (0.027) |
|  |  |  |  |  |  |  |  |  |  |  |  |
|  | | | | | | | | | | | |
| Observations | 4,364 | 4,368 | 4,368 | 4,368 | 4,368 | 4,368 | 4,364 | 4,364 | 4,364 | 4,364 | 4,364 |
| Log Likelihood | -3,366.413 | -5,529.836 | -4,407.735 | -4,575.539 | -5,472.243 | -5,019.253 | -5,197.501 | -5,182.087 | -5,643.273 | -5,680.809 | -4,693.506 |
| Akaike Inf. Crit. | 6,742.825 | 11,069.670 | 8,825.470 | 9,161.078 | 10,954.490 | 10,048.510 | 10,405.000 | 10,374.170 | 11,296.550 | 11,371.620 | 9,397.012 |
| Bayesian Inf. Crit. | 6,774.731 | 11,101.580 | 8,857.381 | 9,192.988 | 10,986.400 | 10,080.420 | 10,436.910 | 10,406.080 | 11,328.450 | 11,403.520 | 9,428.918 |
| Note.^*^p<0.1, ^**^p<0.05, ^***^p<0.01; Coefficients significant according to Benjamini-Hochberg threshold in bold. | | | | | | | | | | | |

**Table A15**

*Motives and negative affect*

|  | | | | | | | | | | | |
| --- | --- | --- | --- | --- | --- | --- | --- | --- | --- | --- | --- |
|  | Dependent variable: | | | | | | | | | | |
|  |  | | | | | | | | | | |
|  | Negative affect | Distressed | Upset | Guilty | Scared | Hostile | Irritable | Ashamed | Nervous | Jittery | Afraid |
|  | (1) | (2) | (3) | (4) | (5) | (6) | (7) | (8) | (9) | (10) | (11) |
|  | | | | | | | | | | | |
| Health motives | -0.008 | -0.005 | 0.010 | -0.002 | -0.011 | 0.007 | **-0.079^***^** | 0.011 | -0.016 | -0.003 | 0.003 |
|  | (0.010) | (0.019) | (0.017) | (0.019) | (0.013) | (0.015) | (0.022) | (0.014) | (0.022) | (0.016) | (0.014) |
|  |  |  |  |  |  |  |  |  |  |  |  |
| Environmental motives | 0.007 | -0.008 | -0.001 | **0.054^***^** | -0.027^**^ | 0.030^**^ | 0.010 | **0.050^***^** | -0.018 | -0.020 | -0.005 |
|  | (0.010) | (0.019) | (0.017) | (0.018) | (0.013) | (0.014) | (0.022) | (0.014) | (0.021) | (0.015) | (0.013) |
|  |  |  |  |  |  |  |  |  |  |  |  |
| Constant | 1.224^***^ | 1.243^***^ | 1.201^***^ | 1.310^***^ | 1.122^***^ | 1.142^***^ | 1.420^***^ | 1.166^***^ | 1.324^***^ | 1.194^***^ | 1.126^***^ |
|  | (0.011) | (0.016) | (0.015) | (0.018) | (0.011) | (0.013) | (0.020) | (0.014) | (0.017) | (0.014) | (0.012) |
|  |  |  |  |  |  |  |  |  |  |  |  |
|  | | | | | | | | | | | |
| Observations | 4,364 | 4,368 | 4,368 | 4,368 | 4,368 | 4,368 | 4,364 | 4,364 | 4,364 | 4,364 | 4,364 |
| Log Likelihood | -996.058 | -3,525.861 | -3,131.986 | -3,622.863 | -1,882.144 | -2,495.397 | -4,303.504 | -2,471.341 | -4,068.504 | -2,802.702 | -2,129.210 |
| Akaike Inf. Crit. | 2,002.116 | 7,061.722 | 6,273.972 | 7,255.726 | 3,774.288 | 5,000.793 | 8,617.009 | 4,952.682 | 8,147.008 | 5,615.404 | 4,268.420 |
| Bayesian Inf. Crit. | 2,034.022 | 7,093.632 | 6,305.882 | 7,287.636 | 3,806.198 | 5,032.703 | 8,648.914 | 4,984.588 | 8,178.914 | 5,647.310 | 4,300.326 |
| Note.^*^p<0.1, ^**^p<0.05, ^***^p<0.01; Coefficients significant according to Benjamini-Hochberg threshold in bold. | | | | | | | | | | | |

**Table A16**

*Meat attachment and perceptions*

|  | | | | | | | | | |
| --- | --- | --- | --- | --- | --- | --- | --- | --- | --- |
|  | Dependent variable: | | | | | | | | |
|  |  | | | | | | | | |
|  | Tolerant  –  Judgmental | Modest  –  Feels superior | Stronger  –  Weaker | Confident  –  Insecure | Moral  –  Immoral | Humble  –  Self-righteous | Inspiring  –  Boring | Preachy  –  More lenient | Stupid  –  Intelligent |
|  | (1) | (2) | (3) | (4) | (5) | (6) | (7) | (8) | (9) |
|  | | | | | | | | | |
| Meat attachment | **0.251^***^** | **0.157^***^** | **0.281^***^** | **0.278^***^** | **0.239^***^** | **0.259^***^** | **0.422^***^** | -**0.187^***^** | **-0.245^***^** |
|  | (0.027) | (0.023) | (0.025) | (0.029) | (0.028) | (0.027) | (0.028) | (0.024) | (0.022) |
|  |  |  |  |  |  |  |  |  |  |
| Constant | 3.203^***^ | 3.630^***^ | 2.694^***^ | 2.519^***^ | 2.612^***^ | 3.324^***^ | 2.352^***^ | 4.362^***^ | 5.051^***^ |
|  | (0.094) | (0.083) | (0.090) | (0.102) | (0.098) | (0.094) | (0.099) | (0.086) | (0.079) |
|  |  |  |  |  |  |  |  |  |  |
|  | | | | | | | | | |
| Observations | 4,334 | 4,334 | 4,334 | 4,334 | 4,334 | 4,334 | 4,334 | 4,334 | 4,334 |
| Log Likelihood | -5,713.769 | -5,147.774 | -5,163.089 | -5,365.604 | -5,267.651 | -5,183.018 | -5,279.432 | -4,922.531 | -4,194.044 |
| Akaike Inf. Crit. | 11,435.540 | 10,303.550 | 10,334.180 | 10,739.210 | 10,543.300 | 10,374.030 | 10,566.860 | 9,853.062 | 8,396.087 |
| Bayesian Inf. Crit. | 11,461.040 | 10,329.050 | 10,359.680 | 10,764.710 | 10,568.800 | 10,399.530 | 10,592.360 | 9,878.559 | 8,421.584 |
|  | | | | | | | | | |
| Note.^*^p<0.1, ^**^p<0.05, ^***^p<0.01; Coefficients significant according to Benjamini-Hochberg threshold in bold.. | | | | | | | | | |

**Table A17**

*Meat attachment and positive affect*

|  | | | | | | | | | | | |
| --- | --- | --- | --- | --- | --- | --- | --- | --- | --- | --- | --- |
|  | Dependent variable: | | | | | | | | | | |
|  |  | | | | | | | | | | |
|  | Positive Affect | Interested | Excited | Strong | Enthusiastic | Proud | Alert | Inspired | Determined | Attentive | Active |
|  | (1) | (2) | (3) | (4) | (5) | (6) | (7) | (8) | (9) | (10) | (11) |
|  | | | | | | | | | | | |
| Meat attachment | **-0.234^***^** | **-0.415^***^** | **-0.221^***^** | -**0.057^**^** | **-0.332^***^** | **-0.230^***^** | **-0.135^***^** | **-0.390^***^** | **-0.167^***^** | **-0.260^***^** | **-0.128^***^** |
|  | (0.026) | (0.031) | (0.028) | (0.028) | (0.032) | (0.031) | (0.030) | (0.030) | (0.030) | (0.033) | (0.030) |
|  |  |  |  |  |  |  |  |  |  |  |  |
| Constant | 2.603^***^ | 3.763^***^ | 2.292^***^ | 1.717^***^ | 2.996^***^ | 2.445^***^ | 2.268^***^ | 3.168^***^ | 2.304^***^ | 2.998^***^ | 2.084^***^ |
|  | (0.091) | (0.110) | (0.098) | (0.101) | (0.114) | (0.111) | (0.106) | (0.108) | (0.106) | (0.118) | (0.107) |
|  |  |  |  |  |  |  |  |  |  |  |  |
|  | | | | | | | | | | | |
| Observations | 4,334 | 4,334 | 4,334 | 4,334 | 4,334 | 4,334 | 4,334 | 4,334 | 4,334 | 4,334 | 4,334 |
| Log Likelihood | -3,304.181 | -5,405.416 | -4,350.121 | -4,529.379 | -5,379.914 | -4,946.637 | -5,148.514 | -5,068.212 | -5,590.655 | -5,609.757 | -4,655.628 |
| Akaike Inf. Crit. | 6,616.361 | 10,818.830 | 8,708.242 | 9,066.758 | 10,767.830 | 9,901.275 | 10,305.030 | 10,144.420 | 11,189.310 | 11,227.510 | 9,319.257 |
| Bayesian Inf. Crit. | 6,641.858 | 10,844.330 | 8,733.739 | 9,092.255 | 10,793.330 | 9,926.772 | 10,330.520 | 10,169.920 | 11,214.810 | 11,253.010 | 9,344.754 |
|  | | | | | | | | | | | |
| Note.^*^p<0.1, ^**^p<0.05, ^***^p<0.01; Coefficients significant according to Benjamini-Hochberg threshold in bold. | | | | | | | | | | | |

**Table A18**

*Meat attachment and negative affect*

|  | | | | | | | | | | | |
| --- | --- | --- | --- | --- | --- | --- | --- | --- | --- | --- | --- |
|  | Dependent variable: | | | | | | | | | | |
|  |  | | | | | | | | | | |
|  | Negative Affect | Distressed | Upset | Guilty | Scared | Hostile | Irritable | Ashamed | Nervous | Jittery | Afraid |
|  | (1) | (2) | (3) | (4) | (5) | (6) | (7) | (8) | (9) | (10) | (11) |
|  | | | | | | | | | | | |
| Meat attachment | **0.029^**^** | **0.069^***^** | **0.091^***^** | **-0.116^***^** | 0.003 | **0.076^***^** | **0.203^***^** | **-0.073^***^** | 0.006 | 0.023 | 0.007 |
|  | (0.012) | (0.016) | (0.015) | (0.018) | (0.011) | (0.013) | (0.021) | (0.014) | (0.017) | (0.015) | (0.012) |
|  |  |  |  |  |  |  |  |  |  |  |  |
| Constant | 1.124^***^ | 1.000^***^ | 0.888^***^ | 1.722^***^ | 1.099^***^ | 0.890^***^ | 0.702^***^ | 1.434^***^ | 1.294^***^ | 1.109^***^ | 1.101^***^ |
|  | (0.041) | (0.057) | (0.055) | (0.066) | (0.037) | (0.048) | (0.074) | (0.051) | (0.062) | (0.054) | (0.041) |
|  |  |  |  |  |  |  |  |  |  |  |  |
|  | | | | | | | | | | | |
| Observations | 4,334 | 4,334 | 4,334 | 4,334 | 4,334 | 4,334 | 4,334 | 4,334 | 4,334 | 4,334 | 4,334 |
| Log Likelihood | -984.075 | -3,490.518 | -3,077.728 | -3,554.053 | -1,870.940 | -2,456.638 | -4,239.307 | -2,443.864 | -4,047.678 | -2,792.877 | -2,113.245 |
| Akaike Inf. Crit. | 1,976.151 | 6,989.037 | 6,163.456 | 7,116.105 | 3,749.879 | 4,921.276 | 8,486.613 | 4,895.728 | 8,103.356 | 5,593.753 | 4,234.490 |
| Bayesian Inf. Crit. | 2,001.648 | 7,014.534 | 6,188.953 | 7,141.602 | 3,775.376 | 4,946.773 | 8,512.110 | 4,921.225 | 8,128.853 | 5,619.250 | 4,259.987 |
|  | | | | | | | | | | | |
| Note.^*^p<0.1, ^**^p<0.05, ^***^p<0.01; Coefficients significant according to Benjamini-Hochberg threshold in bold. | | | | | | | | | | | |

**Table A19**

*Meat attachment, perceptions and diet given*

|  | | | | | | | | | |
| --- | --- | --- | --- | --- | --- | --- | --- | --- | --- |
|  | Dependent variable: | | | | | | | | |
|  |  | | | | | | | | |
|  | Tolerant  –  Judgmental | Modest  –  Feels superior | Stronger  –  Weaker | Confident  –  Insecure | Moral  –  Immoral | Humble  –  Self-righteous | Inspiring  –  Boring | Preachy  –  More lenient | Stupid  –  Intelligent |
|  | (1) | (2) | (3) | (4) | (5) | (6) | (7) | (8) | (9) |
|  | | | | | | | | | |
| Vegetarian | -0.114 | -0.141 | -0.094 | 0.046 | -0.031 | -0.014 | -0.084 | 0.026 | 0.126 |
|  | (0.175) | (0.153) | (0.151) | (0.156) | (0.153) | (0.150) | (0.153) | (0.143) | (0.118) |
|  |  |  |  |  |  |  |  |  |  |
| Meat restrictor | -0.147 | -0.014 | -0.177 | -0.097 | -0.098 | 0.213 | -0.183 | -0.052 | 0.186 |
|  | (0.173) | (0.151) | (0.149) | (0.153) | (0.150) | (0.148) | (0.151) | (0.141) | (0.117) |
|  |  |  |  |  |  |  |  |  |  |
| Meat attachment | **0.213^***^** | **0.136^***^** | **0.265^***^** | **0.262^***^** | **0.230^***^** | **0.289^***^** | **0.410^***^** | **-0.182^***^** | **-0.219^***^** |
|  | (0.047) | (0.042) | (0.042) | (0.045) | (0.044) | (0.043) | (0.044) | (0.040) | (0.035) |
|  |  |  |  |  |  |  |  |  |  |
| Vegetarian: Meat attachment | 0.034 | 0.037 | 0.008 | -0.004 | 0.002 | -0.005 | -0.003 | -0.014 | -0.021 |
|  | (0.049) | (0.043) | (0.042) | (0.044) | (0.043) | (0.042) | (0.043) | (0.040) | (0.033) |
|  |  |  |  |  |  |  |  |  |  |
| Meat restrictor: Meat attachment | 0.055 | 0.015 | 0.031 | 0.041 | 0.018 | -0.063 | 0.032 | 0.001 | -0.040 |
|  | (0.049) | (0.042) | (0.042) | (0.043) | (0.042) | (0.042) | (0.042) | (0.040) | (0.033) |
|  |  |  |  |  |  |  |  |  |  |
| Constant | 3.314^***^ | 3.693^***^ | 2.811^***^ | 2.542^***^ | 2.668^***^ | 3.236^***^ | 2.467^***^ | 4.375^***^ | 4.917^***^ |
|  | (0.168) | (0.148) | (0.151) | (0.161) | (0.157) | (0.153) | (0.157) | (0.143) | (0.123) |
|  |  |  |  |  |  |  |  |  |  |
|  | | | | | | | | | |
| Observations | 4,334 | 4,334 | 4,334 | 4,334 | 4,334 | 4,334 | 4,334 | 4,334 | 4,334 |
| Log Likelihood | -5,711.831 | -5,145.082 | -5,160.349 | -5,363.662 | -5,266.869 | -5,179.849 | -5,275.145 | -4,921.081 | -4,191.161 |
| Akaike Inf. Crit. | 11,439.660 | 10,306.160 | 10,336.700 | 10,743.330 | 10,549.740 | 10,375.700 | 10,566.290 | 9,858.161 | 8,398.322 |
| Bayesian Inf. Crit. | 11,490.660 | 10,357.160 | 10,387.690 | 10,794.320 | 10,600.730 | 10,426.690 | 10,617.280 | 9,909.155 | 8,449.316 |
|  | | | | | | | | | |
| Note.^*^p<0.1, ^**^p<0.05, ^***^p<0.01; Coefficients significant according to Benjamini-Hochberg threshold in bold. | | | | | | | | | |

**Table A20**

*Meat attachment, positive affect and diet given*

|  | | | | | | | | | | | |
| --- | --- | --- | --- | --- | --- | --- | --- | --- | --- | --- | --- |
|  | Dependent variable: | | | | | | | | | | |
|  |  | | | | | | | | | | |
|  | Positive Affect | Interested | Excited | Strong | Enthusiastic | Proud | Alert | Inspired | Determined | Attentive | Active |
|  | (1) | (2) | (3) | (4) | (5) | (6) | (7) | (8) | (9) | (10) | (11) |
|  | | | | | | | | | | | |
| Vegetarian | 0.083 | 0.295^*^ | -0.119 | 0.111 | 0.167 | 0.230^*^ | 0.137 | -0.064 | -0.013 | 0.210 | 0.145 |
|  | (0.087) | (0.154) | (0.117) | (0.122) | (0.152) | (0.135) | (0.145) | (0.140) | (0.164) | (0.160) | (0.125) |
|  |  |  |  |  |  |  |  |  |  |  |  |
| Meat restrictor | 0.032 | 0.216 | -0.101 | 0.081 | 0.052 | 0.188 | -0.006 | 0.178 | 0.038 | -0.037 | 0.024 |
|  | (0.086) | (0.152) | (0.115) | (0.120) | (0.150) | (0.133) | (0.143) | (0.138) | (0.162) | (0.158) | (0.123) |
|  |  |  |  |  |  |  |  |  |  |  |  |
| Meat attachment | **-0.228^***^** | **-0.377^***^** | **-0.241^***^** | -0.040 | **-0.313^***^** | **-0.193^***^** | **-0.125^***^** | **-0.391^***^** | **-0.166^***^** | **-0.246^***^** | **-0.116^***^** |
|  | (0.032) | (0.046) | (0.038) | (0.039) | (0.047) | (0.043) | (0.044) | (0.044) | (0.047) | (0.049) | (0.041) |
|  |  |  |  |  |  |  |  |  |  |  |  |
| Vegetarian: Meat attachment | -0.013 | -0.060 | 0.028 | -0.022 | -0.039 | -0.047 | -0.029 | 0.028 | 0.005 | -0.046 | -0.032 |
|  | (0.025) | (0.043) | (0.033) | (0.034) | (0.043) | (0.038) | (0.041) | (0.039) | (0.046) | (0.045) | (0.035) |
|  |  |  |  |  |  |  |  |  |  |  |  |
| Meat restrictor: Meat attachment | -0.001 | -0.031 | 0.018 | -0.018 | -0.006 | -0.042 | 0.006 | -0.023 | -0.006 | 0.011 | 0.001 |
|  | (0.024) | (0.043) | (0.032) | (0.034) | (0.042) | (0.037) | (0.040) | (0.039) | (0.046) | (0.044) | (0.034) |
|  |  |  |  |  |  |  |  |  |  |  |  |
| Constant | 2.555^***^ | 3.544^***^ | 2.386^***^ | 1.635^***^ | 2.903^***^ | 2.268^***^ | 2.214^***^ | 3.114^***^ | 2.292^***^ | 2.928^***^ | 2.013^***^ |
|  | (0.115) | (0.165) | (0.135) | (0.140) | (0.166) | (0.154) | (0.157) | (0.156) | (0.169) | (0.175) | (0.146) |
|  |  |  |  |  |  |  |  |  |  |  |  |
|  | | | | | | | | | | | |
| Observations | 4,334 | 4,334 | 4,334 | 4,334 | 4,334 | 4,334 | 4,334 | 4,334 | 4,334 | 4,334 | 4,334 |
| Log Likelihood | -3,302.198 | -5,399.565 | -4,348.691 | -4,528.346 | -5,378.660 | -4,943.440 | -5,146.918 | -5,059.433 | -5,590.428 | -5,605.694 | -4,653.857 |
| Akaike Inf. Crit. | 6,620.397 | 10,815.130 | 8,713.381 | 9,072.693 | 10,773.320 | 9,902.879 | 10,309.840 | 10,134.870 | 11,196.850 | 11,227.390 | 9,323.714 |
| Bayesian Inf. Crit. | 6,671.391 | 10,866.120 | 8,764.375 | 9,123.687 | 10,824.320 | 9,953.873 | 10,360.830 | 10,185.860 | 11,247.850 | 11,278.380 | 9,374.708 |
|  | | | | | | | | | | | |
| Note.^*^p<0.1, ^**^p<0.05, ^***^p<0.01; Coefficients significant according to Benjamini-Hochberg threshold in bold. | | | | | | | | | | | |

**Table A21**

*Meat attachment, negative affect and diet given*

|  | | | | | | | | | | | |
| --- | --- | --- | --- | --- | --- | --- | --- | --- | --- | --- | --- |
|  | Dependent variable: | | | | | | | | | | |
|  |  | | | | | | | | | | |
|  | Negative Affect | Distressed | Upset | Guilty | Scared | Hostile | Irritable | Ashamed | Nervous | Jittery | Afraid |
|  | (1) | (2) | (3) | (4) | (5) | (6) | (7) | (8) | (9) | (10) | (11) |
|  | | | | | | | | | | | |
| Vegetarian | 0.021 | 0.030 | 0.069 | 0.021 | 0.046 | -0.075 | -0.016 | 0.207^***^ | -0.065 | 0.009 | -0.007 |
|  | (0.055) | (0.105) | (0.094) | (0.103) | (0.073) | (0.081) | (0.122) | (0.079) | (0.120) | (0.087) | (0.076) |
|  |  |  |  |  |  |  |  |  |  |  |  |
| Meat restrictor | -0.031 | -0.065 | 0.004 | 0.038 | -0.029 | -0.159^**^ | -0.100 | 0.170^**^ | -0.095 | -0.068 | -0.048 |
|  | (0.054) | (0.103) | (0.093) | (0.102) | (0.072) | (0.080) | (0.120) | (0.078) | (0.118) | (0.086) | (0.075) |
|  |  |  |  |  |  |  |  |  |  |  |  |
| Meat attachment | 0.024 | 0.065^**^ | **0.092^***^** | **-0.117^***^** | 0.007 | 0.038^*^ | **0.185^***^** | -0.031 | -0.008 | 0.015 | -0.008 |
|  | (0.017) | (0.028) | (0.026) | (0.030) | (0.019) | (0.023) | (0.034) | (0.023) | (0.032) | (0.025) | (0.021) |
|  |  |  |  |  |  |  |  |  |  |  |  |
| Vegetarian: Meat attachment | -0.002 | -0.006 | -0.013 | 0.003 | -0.014 | 0.029 | 0.003 | -0.056^**^ | 0.014 | -0.001 | 0.015 |
|  | (0.015) | (0.029) | (0.026) | (0.029) | (0.020) | (0.023) | (0.034) | (0.022) | (0.034) | (0.024) | (0.021) |
|  |  |  |  |  |  |  |  |  |  |  |  |
| Meat restrictor: Meat attachment | 0.013 | 0.015 | 0.009 | -0.0003 | 0.005 | 0.058^***^ | 0.037 | -0.043^*^ | 0.018 | 0.019 | 0.020 |
|  | (0.015) | (0.029) | (0.026) | (0.029) | (0.020) | (0.022) | (0.034) | (0.022) | (0.033) | (0.024) | (0.021) |
|  |  |  |  |  |  |  |  |  |  |  |  |
| Constant | 1.129^***^ | 1.017^***^ | 0.857^***^ | 1.696^***^ | 1.093^***^ | 0.991^***^ | 0.752^***^ | 1.272^***^ | 1.363^***^ | 1.136^***^ | 1.126^***^ |
|  | (0.060) | (0.101) | (0.093) | (0.105) | (0.069) | (0.080) | (0.122) | (0.082) | (0.114) | (0.088) | (0.073) |
|  |  |  |  |  |  |  |  |  |  |  |  |
|  | | | | | | | | | | | |
| Observations | 4,334 | 4,334 | 4,334 | 4,334 | 4,334 | 4,334 | 4,334 | 4,334 | 4,334 | 4,334 | 4,334 |
| Log Likelihood | -982.304 | -3,489.084 | -3,075.623 | -3,552.769 | -1,869.515 | -2,449.999 | -4,236.663 | -2,440.051 | -4,046.792 | -2,791.927 | -2,109.063 |
| Akaike Inf. Crit. | 1,980.607 | 6,994.168 | 6,167.246 | 7,121.538 | 3,755.030 | 4,915.998 | 8,489.325 | 4,896.101 | 8,109.584 | 5,599.853 | 4,234.125 |
| Bayesian Inf. Crit. | 2,031.601 | 7,045.162 | 6,218.240 | 7,172.532 | 3,806.024 | 4,966.992 | 8,540.319 | 4,947.095 | 8,160.578 | 5,650.847 | 4,285.119 |
|  | | | | | | | | | | | |
| Note.^*^p<0.1, ^**^p<0.05, ^***^p<0.01; Coefficients significant according to Benjamini-Hochberg threshold in bold. | | | | | | | | | | | |

**Table A22**

*Meat attachment, perceptions and motives*

|  | | | | | | | | | |
| --- | --- | --- | --- | --- | --- | --- | --- | --- | --- |
|  | Dependent variable: | | | | | | | | |
|  |  | | | | | | | | |
|  | Tolerant  –  Judgmental | Modest  –  Feels superior | Stronger  –  Weaker | Confident  –  Insecure | Moral  –  Immoral | Humble  –  Self-righteous | Inspiring  –  Boring | Preachy  –  More lenient | Stupid  –  Intelligent |
|  | (1) | (2) | (3) | (4) | (5) | (6) | (7) | (8) | (9) |
|  | | | | | | | | | |
| Health | -0.011 | 0.009 | 0.024 | 0.267^**^ | **0.298^**^** | 0.177 | 0.126 | -0.148 | **0.238^**^** |
|  | (0.137) | (0.119) | (0.119) | (0.123) | (0.119) | (0.117) | (0.120) | (0.112) | (0.093) |
|  |  |  |  |  |  |  |  |  |  |
| Environmental | -0.121 | -0.168 | -0.182 | -0.040 | **-0.312^***^** | -0.048 | **-0.345^***^** | ­­­ | **0.386^***^** |
|  | (0.133) | (0.116) | (0.116) | (0.119) | (0.116) | (0.114) | (0.117) | (0.109) | (0.090) |
|  |  |  |  |  |  |  |  |  |  |
| Meat attachment | **0.240^***^** | **0.135^***^** | **0.275^***^** | **0.291^***^** | **0.252^***^** | **0.267^***^** | **0.412^***^** | **-0.193^***^** | **-0.207^***^** |
|  | (0.032) | (0.028) | (0.030) | (0.033) | (0.032) | (0.031) | (0.032) | (0.028) | (0.025) |
|  |  |  |  |  |  |  |  |  |  |
| Health: Meat attachment | -0.042 | -0.027 | -0.019 | -0.061^*^ | **-0.080^**^** | **-0.083^**^** | -0.057^*^ | 0.065^**^ | -0.041 |
|  | (0.038) | (0.034) | (0.033) | (0.034) | (0.033) | (0.033) | (0.034) | (0.031) | (0.026) |
|  |  |  |  |  |  |  |  |  |  |
| Environmental: Meat attachment | 0.074^**^ | **0.099^***^** | 0.038 | 0.012 | 0.030 | 0.051 | **0.087^***^** | -0.039 | **-0.090^***^** |
|  | (0.037) | (0.033) | (0.033) | (0.034) | (0.033) | (0.032) | (0.033) | (0.031) | (0.025) |
|  |  |  |  |  |  |  |  |  |  |
| Constant | 3.244^***^ | 3.680^***^ | 2.741^***^ | 2.458^***^ | 2.621^***^ | 3.293^***^ | 2.421^***^ | 4.399^***^ | 4.872^***^ |
|  | (0.114) | (0.100) | (0.106) | (0.117) | (0.113) | (0.109) | (0.113) | (0.100) | (0.090) |
|  |  |  |  |  |  |  |  |  |  |
|  | | | | | | | | | |
| Observations | 4,334 | 4,334 | 4,334 | 4,334 | 4,334 | 4,334 | 4,334 | 4,334 | 4,334 |
| Log Likelihood | -5,672.875 | -5,101.445 | -5,159.536 | -5,361.125 | -5,224.127 | -5,142.041 | -5,267.748 | -4,888.208 | -4,175.810 |
| Akaike Inf. Crit. | 11,361.750 | 10,218.890 | 10,335.070 | 10,738.250 | 10,464.250 | 10,300.080 | 10,551.500 | 9,792.415 | 8,367.621 |
| Bayesian Inf. Crit. | 11,412.740 | 10,269.880 | 10,386.070 | 10,789.240 | 10,515.250 | 10,351.080 | 10,602.490 | 9,843.409 | 8,418.615 |
|  | | | | | | | | | |
| Note.^*^p<0.1, ^**^p<0.05, ^***^p<0.01; Coefficients significant according to Benjamini-Hochberg threshold in bold. | | | | | | | | | |

**Table A23**

*Meat attachment, positive affect and motives*

|  | | | | | | | | | | | |
| --- | --- | --- | --- | --- | --- | --- | --- | --- | --- | --- | --- |
|  | Dependent variable: | | | | | | | | | | |
|  |  | | | | | | | | | | |
|  | Positive Affect | Interested | Excited | Strong | Enthusiastic | Proud | Alert | Inspired | Determined | Attentive | Active |
|  | (1) | (2) | (3) | (4) | (5) | (6) | (7) | (8) | (9) | (10) | (11) |
|  | | | | | | | | | | | |
| Health | 0.007 | 0.014 | -0.057 | -0.024 | -0.021 | 0.00005 | 0.146 | -0.262^**^ | 0.194 | 0.013 | 0.046 |
|  | (0.069) | (0.121) | (0.092) | (0.096) | (0.120) | (0.106) | (0.114) | (0.110) | (0.129) | (0.126) | (0.098) |
|  |  |  |  |  |  |  |  |  |  |  |  |
| Environmental | 0.112^*^ | 0.274^**^ | -0.037 | 0.158^*^ | 0.029 | 0.171^*^ | 0.126 | 0.157 | 0.097 | 0.109 | 0.128 |
|  | (0.067) | (0.118) | (0.089) | (0.094) | (0.116) | (0.103) | (0.111) | (0.107) | (0.126) | (0.123) | (0.095) |
|  |  |  |  |  |  |  |  |  |  |  |  |
| Meat attachment | **-0.227^***^** | **-0.403^***^** | **-0.228^***^** | -0.050 | **-0.332^***^** | **-0.223^***^** | **-0.115^***^** | **-0.400^***^** | **-0.145^***^** | **-0.251^***^** | **-0.115^***^** |
|  | (0.027) | (0.035) | (0.030) | (0.031) | (0.036) | (0.034) | (0.033) | (0.034) | (0.034) | (0.037) | (0.033) |
|  |  |  |  |  |  |  |  |  |  |  |  |
| Health: Meat attachment | 0.006 | 0.025 | 0.010 | 0.015 | 0.012 | 0.011 | -0.035 | 0.075^**^ | -0.054 | 0.006 | -0.014 |
|  | (0.019) | (0.034) | (0.026) | (0.027) | (0.034) | (0.030) | (0.032) | (0.031) | (0.036) | (0.036) | (0.028) |
|  |  |  |  |  |  |  |  |  |  |  |  |
| Environmental: Meat attachment | -0.026 | -0.065^*^ | 0.014 | -0.038 | -0.010 | -0.035 | -0.036 | -0.034 | -0.024 | -0.033 | -0.032 |
|  | (0.019) | (0.033) | (0.025) | (0.026) | (0.033) | (0.029) | (0.031) | (0.030) | (0.035) | (0.035) | (0.027) |
|  |  |  |  |  |  |  |  |  |  |  |  |
| Constant | 2.568^***^ | 3.677^***^ | 2.318^***^ | 1.676^***^ | 2.993^***^ | 2.395^***^ | 2.191^***^ | 3.192^***^ | 2.223^***^ | 2.962^***^ | 2.033^***^ |
|  | (0.096) | (0.124) | (0.107) | (0.111) | (0.127) | (0.121) | (0.118) | (0.120) | (0.122) | (0.132) | (0.117) |
|  |  |  |  |  |  |  |  |  |  |  |  |
|  | | | | | | | | | | | |
| Observations | 4,334 | 4,334 | 4,334 | 4,334 | 4,334 | 4,334 | 4,334 | 4,334 | 4,334 | 4,334 | 4,334 |
| Log Likelihood | -3,301.062 | -5,395.668 | -4,348.947 | -4,526.447 | -5,379.185 | -4,943.012 | -5,147.153 | -5,061.237 | -5,589.423 | -5,608.095 | -4,654.467 |
| Akaike Inf. Crit. | 6,618.124 | 10,807.340 | 8,713.894 | 9,068.895 | 10,774.370 | 9,902.023 | 10,310.310 | 10,138.470 | 11,194.840 | 11,232.190 | 9,324.935 |
| Bayesian Inf. Crit. | 6,669.118 | 10,858.330 | 8,764.888 | 9,119.889 | 10,825.360 | 9,953.017 | 10,361.300 | 10,189.470 | 11,245.840 | 11,283.180 | 9,375.929 |
|  | | | | | | | | | | | |
| Note.^*^p<0.1, ^**^p<0.05, ^***^p<0.01; Coefficients significant according to Benjamini-Hochberg threshold in bold. | | | | | | | | | | | |

**Table A24**

*Meat attachment, negative affect and motives*

|  | | | | | | | | | | | |
| --- | --- | --- | --- | --- | --- | --- | --- | --- | --- | --- | --- |
|  | Dependent variable: | | | | | | | | | | |
|  |  | | | | | | | | | | |
|  | Negative Affect | Distressed | Upset | Guilty | Scared | Hostile | Irritable | Ashamed | Nervous | Jittery | Afraid |
|  | (1) | (2) | (3) | (4) | (5) | (6) | (7) | (8) | (9) | (10) | (11) |
|  | | | | | | | | | | | |
| Health | 0.018 | 0.023 | -0.013 | -0.026 | 0.024 | 0.021 | -0.037 | 0.098 | 0.076 | -0.034 | 0.054 |
|  | (0.043) | (0.083) | (0.074) | (0.081) | (0.057) | (0.064) | (0.096) | (0.063) | (0.095) | (0.068) | (0.060) |
|  |  |  |  |  |  |  |  |  |  |  |  |
| Environmental | 0.008 | -0.010 | 0.005 | 0.211^***^ | -0.048 | -0.096 | -0.126 | **0.186^***^** | 0.057 | -0.002 | -0.083 |
|  | (0.042) | (0.080) | (0.072) | (0.079) | (0.056) | (0.062) | (0.093) | (0.061) | (0.092) | (0.067) | (0.058) |
|  |  |  |  |  |  |  |  |  |  |  |  |
| Meat attachment | 0.031^**^ | **0.071^***^** | **0.090^***^** | **-0.104^***^** | 0.004 | **0.066^***^** | **0.195^***^** | **-0.054^***^** | 0.020 | 0.022 | 0.004 |
|  | (0.013) | (0.019) | (0.018) | (0.021) | (0.013) | (0.016) | (0.024) | (0.017) | (0.021) | (0.018) | (0.014) |
|  |  |  |  |  |  |  |  |  |  |  |  |
| Health: Meat Attachment | -0.008 | -0.008 | 0.006 | 0.007 | -0.010 | -0.005 | -0.013 | -0.025 | -0.027 | 0.009 | -0.015 |
|  | (0.012) | (0.023) | (0.021) | (0.023) | (0.016) | (0.018) | (0.027) | (0.018) | (0.027) | (0.019) | (0.017) |
|  |  |  |  |  |  |  |  |  |  |  |  |
| Environment: Meat attachment | -0.0004 | 0.0001 | -0.002 | -0.046^**^ | 0.006 | 0.036^**^ | 0.039 | -0.040^**^ | -0.021 | -0.005 | 0.023 |
|  | (0.012) | (0.023) | (0.020) | (0.022) | (0.016) | (0.017) | (0.026) | (0.017) | (0.026) | (0.019) | (0.016) |
|  |  |  |  |  |  |  |  |  |  |  |  |
| Constant | 1.117^***^ | 0.997^***^ | 0.890^***^ | 1.667^***^ | 1.106^***^ | 0.914^***^ | 0.750^***^ | 1.352^***^ | 1.257^***^ | 1.119^***^ | 1.111^***^ |
|  | (0.046) | (0.069) | (0.065) | (0.076) | (0.046) | (0.056) | (0.086) | (0.059) | (0.076) | (0.063) | (0.050) |
|  |  |  |  |  |  |  |  |  |  |  |  |
|  | | | | | | | | | | | |
| Observations | 4,334 | 4,334 | 4,334 | 4,334 | 4,334 | 4,334 | 4,334 | 4,334 | 4,334 | 4,334 | 4,334 |
| Log Likelihood | -982.877 | -3,490.296 | -3,077.479 | -3,546.245 | -1,868.445 | -2,451.673 | -4,228.446 | -2,435.179 | -4,046.621 | -2,791.784 | -2,110.980 |
| Akaike Inf. Crit. | 1,981.754 | 6,996.592 | 6,170.958 | 7,108.490 | 3,752.890 | 4,919.345 | 8,472.892 | 4,886.359 | 8,109.243 | 5,599.567 | 4,237.959 |
| Bayesian Inf. Crit. | 2,032.748 | 7,047.586 | 6,221.952 | 7,159.484 | 3,803.884 | 4,970.339 | 8,523.886 | 4,937.353 | 8,160.237 | 5,650.561 | 4,288.953 |
|  | | | | | | | | | | | |
| Note.^*^p<0.1, ^**^p<0.05, ^***^p<0.01; Coefficients significant according to Benjamini-Hochberg threshold in bold. | | | | | | | | | | | |

**PART III. Exploratory analyses**

**Table A25**

*Perceptions and location*

|  | | | | | | | | | |
| --- | --- | --- | --- | --- | --- | --- | --- | --- | --- |
|  | Dependent variable: | | | | | | | | |
|  |  | | | | | | | | |
|  | Tolerant  –  Judgmental | Modest  –  Feels superior | Stronger  –  Weaker | Confident  –  Insecure | Moral  –  Immoral | Humble  –  Self-righteous | Inspiring  –  Boring | Preachy  –  More lenient | Stupid  –  Intelligent |
|  | (1) | (2) | (3) | (4) | (5) | (6) | (7) | (8) | (9) |
|  | | | | | | | | | |
| Refence category: Dinner party at home | | | | | | | | | |
| Pub | -0.075^**^ | -0.022 | -0.054^*^ | -0.032 | -0.026 | -0.061^**^ | -0.042 | **0.108^***^** | 0.010 |
|  | (0.033) | (0.029) | (0.028) | (0.029) | (0.028) | (0.028) | (0.028) | (0.027) | (0.022) |
|  |  |  |  |  |  |  |  |  |  |
| Restaurant | -0.087^***^ | -0.016 | -0.053^*^ | -0.062^**^ | -0.017 | -0.009 | -0.043 | 0.044^*^ | 0.040^*^ |
|  | (0.033) | (0.029) | (0.028) | (0.029) | (0.028) | (0.028) | (0.028) | (0.027) | (0.022) |
|  |  |  |  |  |  |  |  |  |  |
| BBQ | 0.045 | -0.042 | -0.004 | 0.043 | 0.072^**^ | -0.026 | -0.005 | **0.104^***^** | 0.007 |
|  | (0.034) | (0.029) | (0.029) | (0.029) | (0.029) | (0.029) | (0.029) | (0.027) | (0.022) |
|  |  |  |  |  |  |  |  |  |  |
| Constant | 4.101^***^ | 4.194^***^ | 3.692^***^ | 3.492^***^ | 3.433^***^ | 4.245^***^ | 3.835^***^ | 3.648^***^ | 4.189^***^ |
|  | (0.030) | (0.026) | (0.028) | (0.030) | (0.029) | (0.028) | (0.030) | (0.026) | (0.023) |
|  |  |  |  |  |  |  |  |  |  |
|  | | | | | | | | | |
| Observations | 4,362 | 4,362 | 4,362 | 4,362 | 4,362 | 4,362 | 4,362 | 4,362 | 4,362 |
| Log Likelihood | -5,784.971 | -5,205.181 | -5,257.446 | -5,455.003 | -5,340.908 | -5,260.104 | -5,422.292 | -4,969.497 | -4,281.434 |
| Akaike Inf. Crit. | 11,581.940 | 10,422.360 | 10,526.890 | 10,922.010 | 10,693.820 | 10,532.210 | 10,856.580 | 9,950.993 | 8,574.868 |
| Bayesian Inf. Crit. | 11,620.230 | 10,460.650 | 10,565.180 | 10,960.290 | 10,732.100 | 10,570.490 | 10,894.870 | 9,989.277 | 8,613.152 |
|  | | | | | | | | | |
| Note. | ^*^p<0.1, ^**^p<0.05, ^***^p<0.01; Coefficients significant according to Benjamini-Hochberg threshold in bold. | | | | | | | | |

**Table A26**

*Positive affect and location*

|  | | | | | | | | | | | |
| --- | --- | --- | --- | --- | --- | --- | --- | --- | --- | --- | --- |
|  | Dependent variable: | | | | | | | | | | |
|  |  | | | | | | | | | | |
|  | Positive Affect | Interested | Excited | Strong | Enthusiastic | Proud | Alert | Inspired | Determined | Attentive | Active |
|  | (1) | (2) | (3) | (4) | (5) | (6) | (7) | (8) | (9) | (10) | (11) |
|  | | | | | | | | | | | |
| Reference category: Dinner party at home | | | | | | | | | | | |
| Pub | **-0.254^***^** | **-0.213^***^** | **-0.173^***^** | **-0.157^***^** | **-0.273^***^** | -0.016 | **-0.306^***^** | **-0.207^***^** | **-0.523^***^** | **-0.380^***^** | **-0.293^***^** |
|  | (0.015) | (0.028) | (0.021) | (0.022) | (0.027) | (0.025) | (0.026) | (0.025) | (0.028) | (0.028) | (0.028) |
|  |  |  |  |  |  |  |  |  |  |  |  |
| Restaurant | **-0.234^***^** | **-0.191^***^** | **-0.090^***^** | **-0.154^***^** | **-0.234^***^** | -0.012 | **-0.279^***^** | **-0.187^***^** | **-0.545^***^** | **-0.369^***^** | **-0.276^***^** |
|  | (0.015) | (0.028) | (0.021) | (0.022) | (0.027) | (0.025) | (0.026) | (0.025) | (0.028) | (0.028) | (0.022) |
|  |  |  |  |  |  |  |  |  |  |  |  |
| BBQ | -0.010 | 0.017 | 0.024 | -0.045^**^ | 0.001 | **0.069^***^** | -0.041 | -0.030 | -0.057^*^ | 0.014 | 0.025* |
|  | (0.015) | (0.029) | (0.021) | (0.022) | (0.028) | (0.025) | (0.026) | (0.026) | (0.028) | (0.029) | (0.022) |
|  |  |  |  |  |  |  |  |  |  |  |  |
| Constant | 1.921^***^ | 2.422^***^ | 1.587^***^ | 1.611^***^ | 1.975^***^ | 1.644^***^ | 1.960^***^ | 1.926^***^ | 2.012^***^ | 2.29^***^ | 1.587^***^ |
|  | (0.023) | (0.032) | (0.026) | (0.027) | (0.032) | (0.030) | (0.029) | (0.031) | (0.03) | (0.033) | (0.028) |
|  |  |  |  |  |  |  |  |  |  |  |  |
|  | | | | | | | | | | | |
| Observations | 4,364 | 4,368 | 4,368 | 4,368 | 4,368 | 4,368 | 4,364 | 4,364 | 4,364 | 4,364 | 4,364 |
| Log Likelihood | -3,126.645 | -5,480.989 | -4,355.016 | -4,538.810 | -5,387.592 | -5,014.390 | -5,090.319 | -5,132.857 | -5,342.946 | -5,522.016 | -4,544.924 |
| Akaike Inf. Crit. | 6,265.290 | 10,973.980 | 8,722.032 | 9,089.621 | 10,787.180 | 10,040.780 | 10,192.640 | 10,277.720 | 10,697.890 | 11,056.000 | 9,101.847 |
| Bayesian Inf. Crit. | 6,303.577 | 11,012.270 | 8,760.324 | 9,127.913 | 10,825.480 | 10,079.070 | 10,230.920 | 10,316.000 | 10,736.180 | 11,094.320 | 9,140.134 |
|  | | | | | | | | | | | |
| Note. | ^*^p<0.1, ^**^p<0.05, ^***^p<0.01; Coefficients significant according to Benjamini-Hochberg threshold in bold. | | | | | | | | | | |

**Table A27**

*Negative affect and location*

|  | | | | | | | | | | | |
| --- | --- | --- | --- | --- | --- | --- | --- | --- | --- | --- | --- |
|  | Dependent variable: | | | | | | | | | | |
|  |  | | | | | | | | | | |
|  | Negative Affect | Distressed | Upset | Guilty | Scared | Hostile | Irritable | Ashamed | Nervous | Jittery | Afraid |
|  | (1) | (2) | (3) | (4) | (5) | (6) | (7) | (8) | (9) | (10) | (11) |
|  | | | | | | | | | | | |
| Reference category: Dinner party at home | | | | | | | | | | | |
| Pub | **-0.148^***^** | **-0.237^***^** | **-0.119^***^** | **0.053^***^** | **-0.116^***^** | **-0.053^***^** | **-0.318^***^** | 0.022 | **-0.402^***^** | **-0.169^***^** | **-0.137^***^** |
|  | (0.010) | (0.019) | (0.017) | (0.019) | (0.013) | (0.015) | (0.022) | (0.015) | (0.021) | (0.016) | (0.014) |
|  |  |  |  |  |  |  |  |  |  |  |  |
| Restaurant | **-0.143^***^** | **-0.227^***^** | **-0.125^***^** | **0.059^***^** | **-0.120^***^** | **-0.068^***^** | **-0.286^***^** | **0.037^**^** | **-0.398^***^** | **-0.163^***^** | **-0.133^***^** |
|  | (0.010) | (0.019) | (0.017) | (0.019) | (0.013) | (0.015) | (0.022) | (0.015) | (0.021) | (0.016) | (0.014) |
|  |  |  |  |  |  |  |  |  |  |  |  |
| BBQ | **-0.063^***^** | **-0.124^***^** | **-0.075^***^** | 0.002 | **-0.046^***^** | -0.029^*^ | **-0.117^***^** | -0.005 | **-0.134^***^** | **-0.045^***^** | **-0.059^***^** |
|  | (0.010) | (0.019) | (0.018) | (0.019) | (0.014) | (0.015) | (0.022) | (0.015) | (0.021) | (0.016) | (0.014) |
|  |  |  |  |  |  |  |  |  |  |  |  |
| Constant | 1.313^***^ | 1.387^***^ | 1.283^***^ | 1.297^***^ | 1.182^***^ | 1.191^***^ | 1.582^***^ | 1.170^***^ | 1.550^***^ | 1.283^***^ | 1.208^***^ |
|  | (0.011) | (0.018) | (0.017) | (0.019) | (0.012) | (0.014) | (0.022) | (0.015) | (0.019) | (0.016) | (0.013) |
|  |  |  |  |  |  |  |  |  |  |  |  |
|  | | | | | | | | | | | |
| Observations | 4,364 | 4,368 | 4,368 | 4,368 | 4,368 | 4,368 | 4,364 | 4,364 | 4,364 | 4,364 | 4,368 |
| Log Likelihood | -845.221 | -3,428.600 | -3,099.614 | -3,620.159 | -1,829.736 | -2,485.978 | -4,178.224 | -2,472.530 | -3,818.972 | -845.221 | -2065.400 |
| Akaike Inf. Crit. | 1,702.442 | 6,869.201 | 6,211.229 | 7,252.317 | 3,671.472 | 4,983.957 | 8,368.447 | 4,957.059 | 7,649.943 | 1,702.442 | 4142.900 |
| Bayesian Inf. Crit. | 1,740.729 | 6,907.493 | 6,249.521 | 7,290.610 | 3,709.764 | 5,022.249 | 8,406.734 | 4,995.346 | 7,688.230 | 1,740.729 | 4181.200 |
|  | | | | | | | | | | | |
| Note. | ^*^p<0.1, ^**^p<0.05, ^***^p<0.01; Coefficients significant according to Benjamini-Hochberg threshold in bold. | | | | | | | | | | |

**Table A28**

*Perceptions and catering responsibility*

|  | | | | | | | | | |
| --- | --- | --- | --- | --- | --- | --- | --- | --- | --- |
|  | Dependent variable: | | | | | | | | |
|  |  | | | | | | | | |
|  | Tolerant  –  Judgmental | Modest  –  Feels superior | Stronger  –  Weaker | Confident  –  Insecure | Moral  –  Immoral | Humble  –  Self-righteous | Inspiring  –  Boring | Preachy  –  More lenient | Stupid  –  Intelligent |
|  | (1) | (2) | (3) | (4) | (5) | (6) | (7) | (8) | (9) |
|  | | | | | | | | | |
| Refence category: Not catering | | | | | | | | | |
| Catering | **0.103^***^** | -0.001 | **0.052^***^** | **0.068^***^** | **0.056^***^** | 0.023 | 0.040^**^ | -0.026 | -0.022 |
|  | (0.023) | (0.021) | (0.020) | (0.021) | (0.020) | (0.020) | (0.020) | (0.019) | (0.016) |
|  |  |  |  |  |  |  |  |  |  |
| Constant | 4.021^***^ | 4.175^***^ | 3.638^***^ | 3.445^***^ | 3.411^***^ | 4.210^***^ | 3.792^***^ | 3.724^***^ | 4.214^***^ |
|  | (0.025) | (0.022) | (0.024) | (0.026) | (0.025) | (0.024) | (0.027) | (0.022) | (0.020) |
|  |  |  |  |  |  |  |  |  |  |
|  | | | | | | | | | |
| Observations | 4,362 | 4,362 | 4,362 | 4,362 | 4,362 | 4,362 | 4,362 | 4,362 | 4,362 |
| Log Likelihood | -5,785.946 | -5,206.230 | -5,257.454 | -5,456.637 | -5,344.054 | -5,262.205 | -5,422.306 | -4,979.653 | -4,282.466 |
| Akaike Inf. Crit. | 11,579.890 | 10,420.460 | 10,522.910 | 10,921.270 | 10,696.110 | 10,532.410 | 10,852.610 | 9,967.305 | 8,572.932 |
| Bayesian Inf. Crit. | 11,605.410 | 10,445.980 | 10,548.430 | 10,946.800 | 10,721.630 | 10,557.930 | 10,878.140 | 9,992.828 | 8,598.455 |
|  | | | | | | | | | |
| Note. | ^*^p<0.1, ^**^p<0.05, ^***^p<0.01; Coefficients significant according to Benjamini-Hochberg threshold in bold. | | | | | | | | |

**Table A29**

*Positive affect and catering responsibility*

|  | | | | | | | | | | | |
| --- | --- | --- | --- | --- | --- | --- | --- | --- | --- | --- | --- |
|  | Dependent variable: | | | | | | | | | | |
|  |  | | | | | | | | | | |
|  | Positive Affect | Interested | Excited | Strong | Enthusiastic | Proud | Alert | Inspired | Determined | Attentive | Active |
|  | (1) | (2) | (3) | (4) | (5) | (6) | (7) | (8) | (9) | (10) | (11) |
|  | | | | | | | | | | | |
| Reference category: Not catering | | | | | | | | | | | |
| Catering | **0.239^***^** | **0.210^***^** | **0.143^***^** | **0.134^***^** | **0.254^***^** | **0.047^***^** | **0.272^***^** | **0.182^***^** | **0.507^***^** | **0.367^***^** | **0.273^***^** |
|  | (0.010) | (0.020) | (0.015) | (0.016) | (0.019) | (0.018) | (0.018) | (0.018) | (0.020) | (0.020) | (0.015) |
|  |  |  |  |  |  |  |  |  |  |  |  |
| Constant | 1.678^***^ | 2.220^***^ | 1.455^***^ | 1.455^***^ | 1.722^***^ | 1.630^***^ | 1.668^***^ | 1.730^***^ | 1.478^***^ | 1.920^***^ | 1.502^***^ |
|  | (0.022) | (0.029) | (0.024) | (0.024) | (0.029) | (0.027) | (0.026) | (0.028) | (0.026) | (0.030) | (0.026) |
|  |  |  |  |  |  |  |  |  |  |  |  |
|  | | | | | | | | | | | |
| Observations | 4,364 | 4,368 | 4,368 | 4,368 | 4,368 | 4,368 | 4,364 | 4,364 | 4,364 | 4,364 | 4,364 |
| Log Likelihood | -3,127.819 | -5,481.449 | -4,363.526 | -4,540.864 | -5,388.629 | -5,018.158 | -5,092.092 | -5,133.848 | -5,345.287 | -5,522.212 | -4,545.877 |
| Akaike Inf. Crit. | 6,263.638 | 10,970.900 | 8,735.052 | 9,089.727 | 10,785.260 | 10,044.320 | 10,192.180 | 10,275.700 | 10,698.570 | 11,052.420 | 9,099.755 |
| Bayesian Inf. Crit. | 6,289.162 | 10,996.430 | 8,760.580 | 9,115.255 | 10,810.790 | 10,069.840 | 10,217.710 | 10,301.220 | 10,724.100 | 11,077.950 | 9,125.279 |
|  | | | | | | | | | | | |
| Note. | ^*^p<0.1, ^**^p<0.05, ^***^p<0.01; Coefficients significant according to Benjamini-Hochberg threshold in bold. | | | | | | | | | | |

**Table A30**

*Negative affect and catering responsibility*

|  | | | | | | | | | | | |
| --- | --- | --- | --- | --- | --- | --- | --- | --- | --- | --- | --- |
|  | Dependent variable: | | | | | | | | | | |
|  |  | | | | | | | | | | |
|  | Negative Affect | Distressed | Upset | Guilty | Scared | Hostile | Irritable | Ashamed | Nervous | Jittery | Afraid |
|  | (1) | (2) | (3) | (4) | (5) | (6) | (7) | (8) | (9) | (10) | (11) |
|  | | | | | | | | | | | |
| Reference category: Not catering | | | | | | | | | | | |
| Catering | **0.115^***^** | **0.172^***^** | **0.086^***^** | **-0.055^***^** | **0.096^***^** | **0.047^***^** | **0.246^***^** | **-0.032^***^** | **0.335^***^** | **0.145^***^** | **0.107^***^** |
|  | (0.007) | (0.014) | (0.012) | (0.014) | (0.010) | (0.011) | (0.016) | (0.011) | (0.015) | (0.011) | (0.010) |
|  |  |  |  |  |  |  |  |  |  |  |  |
| Constant | 1.168^***^ | 1.155^***^ | 1.161^***^ | 1.353^***^ | 1.064^***^ | 1.130^***^ | 1.280^***^ | 1.199^***^ | 1.150^***^ | 1.117^***^ | 1.073^***^ |
|  | (0.010) | (0.015) | (0.014) | (0.017) | (0.010) | (0.012) | (0.019) | (0.013) | (0.016) | (0.014) | (0.011) |
|  |  |  |  |  |  |  |  |  |  |  |  |
|  | | | | | | | | | | | |
| Observations | 4,364 | 4,368 | 4,368 | 4,368 | 4,368 | 4,368 | 4,364 | 4,364 | 4,364 | 4,364 | 4,364 |
| Log Likelihood | -865.886 | -3,448.920 | -3,108.493 | -3,620.216 | -1,835.384 | -2,488.280 | -4,193.050 | -2,473.124 | -3,838.561 | -2,722.261 | -2,073.965 |
| Akaike Inf. Crit. | 1,739.773 | 6,905.839 | 6,224.985 | 7,248.433 | 3,678.768 | 4,984.560 | 8,394.100 | 4,954.248 | 7,685.123 | 5,452.522 | 4,155.929 |
| Bayesian Inf. Crit. | 1,765.298 | 6,931.368 | 6,250.514 | 7,273.961 | 3,704.296 | 5,010.088 | 8,419.625 | 4,979.772 | 7,710.648 | 5,478.046 | 4,181.454 |
|  | | | | | | | | | | | |
| Note. | ^*^p<0.1, ^**^p<0.05, ^***^p<0.01; Coefficients significant according to Benjamini-Hochberg threshold in bold. | | | | | | | | | | |

**Table A31**

*Perceptions and company*

|  | | | | | | | | | |
| --- | --- | --- | --- | --- | --- | --- | --- | --- | --- |
|  | Dependent variable: | | | | | | | | |
|  |  | | | | | | | | |
|  | Tolerant  –  Judgmental | Modest  –  Feels superior | Stronger  –  Weaker | Confident  –  Insecure | Moral  –  Immoral | Humble  –  Self-righteous | Inspiring  –  Boring | Preachy  –  More lenient | Stupid  –  Intelligent |
|  | (1) | (2) | (3) | (4) | (5) | (6) | (7) | (8) | (9) |
|  | | | | | | | | | |
| Refence category: Colleague | | | | | | | | | |
| Friend | -0.007 | 0.003 | 0.020 | 0.009 | -0.010 | 0.052^*^ | 0.047^*^ | -0.031 | 0.010 |
|  | (0.032) | (0.028) | (0.027) | (0.028) | (0.027) | (0.027) | (0.028) | (0.026) | (0.021) |
|  |  |  |  |  |  |  |  |  |  |
| Family member | 0.056^*^ | 0.012 | 0.032 | 0.047^*^ | 0.007 | 0.048^*^ | 0.032 | -0.019 | -0.008 |
|  | (0.032) | (0.028) | (0.028) | (0.028) | (0.028) | (0.027) | (0.028) | (0.026) | (0.021) |
|  |  |  |  |  |  |  |  |  |  |
| Constant | 4.055^***^ | 4.170^***^ | 3.647^***^ | 3.459^***^ | 3.440^***^ | 4.187^***^ | 3.786^***^ | 3.728^***^ | 4.202^***^ |
|  | (0.029) | (0.025) | (0.027) | (0.029) | (0.028) | (0.027) | (0.030) | (0.025) | (0.023) |
|  |  |  |  |  |  |  |  |  |  |
|  | | | | | | | | | |
| Observations | 4,362 | 4,362 | 4,362 | 4,362 | 4,362 | 4,362 | 4,362 | 4,362 | 4,362 |
| Log Likelihood | -5,793.110 | -5,206.133 | -5,260.117 | -5,460.530 | -5,347.720 | -5,260.593 | -5,422.781 | -4,979.834 | -4,283.035 |
| Akaike Inf. Crit. | 11,596.220 | 10,422.270 | 10,530.230 | 10,931.060 | 10,705.440 | 10,531.190 | 10,855.560 | 9,969.668 | 8,576.071 |
| Bayesian Inf. Crit. | 11,628.120 | 10,454.170 | 10,562.140 | 10,962.960 | 10,737.340 | 10,563.090 | 10,887.470 | 10,001.570 | 8,607.974 |
|  | | | | | | | | | |
| Note. | ^*^p<0.1, ^**^p<0.05, ^***^p<0.01; Coefficients significant according to Benjamini-Hochberg threshold in bold. | | | | | | | | |

**Table A32**

*Positive affect and company*

|  | | | | | | | | | | | |
| --- | --- | --- | --- | --- | --- | --- | --- | --- | --- | --- | --- |
|  | Dependent variable: | | | | | | | | | | |
|  |  | | | | | | | | | | |
|  | Positive Affect | Interested | Excited | Strong | Enthusiastic | Proud | Alert | Inspired | Determined | Attentive | Active |
|  | (1) | (2) | (3) | (4) | (5) | (6) | (7) | (8) | (9) | (10) | (11) |
|  | | | | | | | | | | | |
| Reference category: Colleague | | | | | | | | | | | |
| Friend | 0.012 | -0.046^*^ | 0.001 | -0.008 | 0.006 | 0.034 | 0.010 | 0.052^**^ | 0.033 | 0.006 | 0.012 |
|  | (0.016) | (0.028) | (0.021) | (0.022) | (0.027) | (0.024) | (0.026) | (0.025) | (0.029) | (0.029) | (0.016) |
|  |  |  |  |  |  |  |  |  |  |  |  |
| Family member | 0.003 | -0.006 | 0.002 | -0.014 | 0.004 | **0.086^***^** | -0.031 | 0.023 | -0.022 | -0.003 | 0.003 |
|  | (0.016) | (0.028) | (0.021) | (0.022) | (0.027) | (0.024) | (0.026) | (0.025) | (0.030) | (0.029) | (0.016) |
|  |  |  |  |  |  |  |  |  |  |  |  |
| Constant | 1.790^***^ | 2.341^***^ | 1.524^***^ | 1.528^***^ | 1.843^***^ | 1.613^***^ | 1.808^***^ | 1.794^***^ | 1.722^***^ | 2.099^***^ | 1.790^***^ |
|  | (0.023) | (0.031) | (0.026) | (0.027) | (0.031) | (0.029) | (0.029) | (0.030) | (0.030) | (0.033) | (0.023) |
|  |  |  |  |  |  |  |  |  |  |  |  |
|  | | | | | | | | | | | |
| Observations | 4,364 | 4,368 | 4,368 | 4,368 | 4,368 | 4,368 | 4,364 | 4,364 | 4,364 | 4,364 | 4,364 |
| Log Likelihood | -3,367.863 | -5,534.157 | -4,408.802 | -4,576.532 | -5,472.727 | -5,015.337 | -5,196.622 | -5,181.611 | -5,641.634 | -5,681.857 | -4,692.540 |
| Akaike Inf. Crit. | 6,745.726 | 11,078.310 | 8,827.603 | 9,163.065 | 10,955.450 | 10,040.670 | 10,403.240 | 10,373.220 | 11,293.270 | 11,373.710 | 9,395.080 |
| Bayesian Inf. Crit. | 6,777.631 | 11,110.220 | 8,859.513 | 9,194.975 | 10,987.360 | 10,072.580 | 10,435.150 | 10,405.130 | 11,325.170 | 11,405.620 | 9,426.986 |
|  | | | | | | | | | | | |
| Note. | ^*^p<0.1, ^**^p<0.05, ^***^p<0.01; Coefficients significant according to Benjamini-Hochberg threshold in bold. | | | | | | | | | | |

**Table A33**

*Negative affect and company*

|  | | | | | | | | | | | |
| --- | --- | --- | --- | --- | --- | --- | --- | --- | --- | --- | --- |
|  | Dependent variable: | | | | | | | | | | |
|  |  | | | | | | | | | | |
|  | Negative Affect | Distressed | Upset | Guilty | Scared | Hostile | Irritable | Ashamed | Nervous | Jittery | Afraid |
|  | (1) | (2) | (3) | (4) | (5) | (6) | (7) | (8) | (9) | (10) | (11) |
|  | | | | | | | | | | | |
| Reference category: Colleague | | | | | | | | | | | |
| Friend | -0.007 | -0.004 | 0.014 | -0.012 | 0.010 | 0.007 | 0.016 | -0.026^*^ | -0.032 | -0.012 | -0.005 |
|  | (0.010) | (0.019) | (0.017) | (0.019) | (0.013) | (0.015) | (0.022) | (0.014) | (0.022) | (0.016) | (0.014) |
|  |  |  |  |  |  |  |  |  |  |  |  |
| Family member | -0.013 | 0.004 | 0.034^**^ | -0.044^**^ | -0.006 | 0.004 | 0.015 | -0.037^***^ | -0.058^***^ | -0.008 | -0.021 |
|  | (0.010) | (0.019) | (0.017) | (0.019) | (0.013) | (0.015) | (0.022) | (0.014) | (0.022) | (0.016) | (0.014) |
|  |  |  |  |  |  |  |  |  |  |  |  |
| Constant | 1.231^***^ | 1.240^***^ | 1.188^***^ | 1.344^***^ | 1.109^***^ | 1.150^***^ | 1.390^***^ | 1.205^***^ | 1.344^***^ | 1.195^***^ | 1.134^***^ |
|  | (0.011) | (0.017) | (0.016) | (0.019) | (0.011) | (0.014) | (0.022) | (0.014) | (0.019) | (0.015) | (0.012) |
|  |  |  |  |  |  |  |  |  |  |  |  |
|  | | | | | | | | | | | |
| Observations | 4,364 | 4,368 | 4,368 | 4,368 | 4,368 | 4,368 | 4,364 | 4,364 | 4,364 | 4,364 | 4,364 |
| Log Likelihood | -996.284 | -3,525.857 | -3,130.199 | -3,625.316 | -1,883.542 | -2,497.496 | -4,311.885 | -2,474.252 | -4,065.416 | -2,803.290 | -2,128.156 |
| Akaike Inf. Crit. | 2,002.567 | 7,061.713 | 6,270.399 | 7,260.632 | 3,777.083 | 5,004.993 | 8,633.771 | 4,958.505 | 8,140.832 | 5,616.579 | 4,266.312 |
| Bayesian Inf. Crit. | 2,034.473 | 7,093.624 | 6,302.309 | 7,292.542 | 3,808.994 | 5,036.903 | 8,665.676 | 4,990.411 | 8,172.738 | 5,648.485 | 4,298.217 |
|  | | | | | | | | | | | |
| Note. | ^*^p<0.1, ^**^p<0.05, ^***^p<0.01; Coefficients significant according to Benjamini-Hochberg threshold in bold. | | | | | | | | | | |

**Table A34**

*Comfort and motives and diet*

|  | Dependent variable: | | | |
| --- | --- | --- | --- | --- |
|  |  | | | |
|  | Meat eater comfort | | Meal requester comfort | |
|  | | | | |
| Health | -0.035 |  | -0.019 |  |
|  | (0.028) |  | (0.028) |  |
|  |  |  |  |  |
| Environment | -0.007 |  | **0.086^***^** |  |
|  | (0.027) |  | (0.027) |  |
|  |  |  |  |  |
| Vegetarian |  | 0.001 |  | 0.024 |
|  |  | (0.035) |  | (0.035) |
|  |  |  |  |  |
| Meat restrictor |  | -0.001 |  | 0.015 |
|  |  | (0.035) |  | (0.035) |
|  |  |  |  |  |
| Constant | 1.980^***^ | 1.969^***^ | 1.824^***^ | 1.826^***^ |
|  | (0.029) | (0.038) | (0.031) | (0.040) |
|  |  |  |  |  |
|  | | | | |
| Observations | 4,362 | 4,362 | 4,362 | 4,362 |
| Log Likelihood | -5,473.604 | -5,474.428 | -5,548.789 | -5,555.804 |
| Akaike Inf. Crit. | 10,957.210 | 10,958.860 | 11,107.580 | 11,121.610 |
| Bayesian Inf. Crit. | 10,989.110 | 10,990.760 | 11,139.480 | 11,153.510 |
|  | | | | |
| Note. | ^*^p<0.1, ^**^p<0.05, ^***^p<0.01; Coefficients significant according to Benjamini-Hochberg threshold in bold. | | | |

**Table A35**

*Invite back and motives and diet*

|  | | |
| --- | --- | --- |
|  | Dependent variable: | |
|  |  | |
|  | Invite back | |
|  | | |
| Health | -0.057^**^ |  |
|  | (0.027) |  |
|  |  |  |
| Environment | 0.035 |  |
|  | (0.027) |  |
|  |  |  |
| Vegetarian |  | -0.004 |
|  |  | (0.035) |
|  |  |  |
| Meat restrictor |  | 0.045 |
|  |  | (0.035) |
|  |  |  |
| Constant | 1.744^***^ | 1.720^***^ |
|  | (0.030) | (0.039) |
|  |  |  |
|  | | |
| Observations | 4,362 | 4,362 |
| Log Likelihood | -5,484.097 | -5,486.654 |
| Akaike Inf. Crit. | 10,978.190 | 10,983.310 |
| Bayesian Inf. Crit. | 11,010.100 | 11,015.210 |
| Note.^*^p<0.1, ^**^p<0.05, ^***^p<0.01; Coefficients significant according to Benjamini-Hochberg threshold in bold. | | |
